# The promise of computer adaptive testing in collection of orthopaedic outcomes: an evaluation of PROMIS utilization

**DOI:** 10.1186/s41687-021-00407-w

**Published:** 2022-01-04

**Authors:** Liam H. Wong, James E. Meeker

**Affiliations:** 1 School of Medicine, Oregon Health & Science University, Portland, OR USA; 2 Department of Orthopaedics and Rehabilitation, Oregon Health & Science University, 3303 S. Bond Avenue, Portland, OR 97239 USA

**Keywords:** PROMIS, Orthopaedic patient-reported outcomes, Orthopaedics, Orthopaedic surgery, PROMIS validation, PROMIS use

## Abstract

**Background:**

A crucial component to improving patient care is better clinician understanding of patients’ health-related quality of life (HRQoL). In orthopaedic surgery, HRQoL assessment instruments such as the NIH developed Patient Reported Outcomes Measurement Information System (PROMIS), provide surgeons with a framework to assess how a treatment or medical condition is affecting each patient’s HRQoL. PROMIS has been demonstrated as a valuable instrument in many diseases; however, the extent to which orthopaedic surgery subspecialties have used and validated PROMIS measures in peer-reviewed research is unclear.

**Methods:**

Systematic scoping methodology was used to investigate the characteristics of studies using PROMIS to assess HRQoL measures as orthopaedic surgical outcomes as well as studies validating computerized adaptive test (CAT) PROMIS physical health (PH) domains including: Physical Function (PF), Upper Extremity (UE), Lower Extremity (LE).

**Results:**

A systematic search of PubMed identified 391 publications utilizing PROMIS in orthopaedics; 153 (39%) were PROMIS PH CAT validation publications. One-hundred publications were in Hand and Upper Extremity, 69 in Spine, 44 in Adult Reconstruction, 43 in Foot and Ankle, 43 in Sports, 37 in Trauma, 31 in General orthopaedics, and 24 in Tumor. From 2011 through 2020 there was an upward trend in orthopaedic PROMIS publications each year (range, 1–153) and an increase in studies investigating or utilizing PROMIS PH CAT domains (range, 1–105). Eighty-five percent (n = 130) of orthopaedic surgery PROMIS PH CAT validation publications (n = 153) analyzed PF; 30% (n = 46) analyzed UE; 3% (n = 4) analyzed LE.

**Conclusions:**

PROMIS utilization within orthopaedics as a whole has significantly increased within the past decade, particularly within PROMIS CAT domains. The existing literature reviewed in this scoping study demonstrates that PROMIS PH CAT domains (PF, UE, and LE) are reliable, responsive, and interpretable in most contexts of patient care throughout all orthopaedic surgery subspecialties. The expanded use of PROMIS CATs in orthopaedic surgery highlights the potential for improved quality of patient care. While challenges of integrating PROMIS into electronic medical records exist, expanded use of PROMIS CAT measurement instruments throughout orthopaedic surgery should be performed.

**Plain english summary** In orthopaedic surgery, health-related quality of life tools such as the NIH developed Patient Reported Outcomes Measurement Information System (PROMIS), offer patients an opportunity to better understand their medical condition and be involved in their own care. Additionally, PROMIS provides surgeons with a framework to assess how a treatment or medical condition is affecting each patient’s functional status and quality of life. The efficacy of PROMIS has been demonstrated in many diseases; however, its application throughout orthopaedic care has yet to be depicted. This study sought to identify the extent to which all orthopaedic surgery subspecialties have used and validated PROMIS measures in peer-reviewed research in order to identify its potential as an applicable and valuable tool across specialties. We determined that PROMIS utilization has significantly increased within the past decade. The existing literature reviewed in this scoping study demonstrates that the PROMIS computerized adaptive test domains evaluating physical function status are reliable, responsive, and interpretable in most contexts of patient care throughout all orthopaedic surgery subspecialties. Based on these results, this study recommends the expanded and more uniform use of PROMIS computerized adaptive test measurement instruments in the clinical care of orthopaedic patients.

**Supplementary Information:**

The online version contains supplementary material available at 10.1186/s41687-021-00407-w.

## Introduction

Advances in health information technology have the potential to elevate the quality of patient care, especially by providing clinicians with efficient measures of patient reported outcomes (PROs) that provide insights into health-related quality of life (HRQoL) during treatment. In the field of orthopaedic surgery, HRQoL assessment instruments help elucidate patients’ well-being and functional capabilities beyond visible outcomes [1, 2]. Numerous validated HRQoL assessment instruments exist in orthopaedics, commonly described as legacy measures. These include American Shoulder and Elbow Surgeons Score (ASES), Disabilities of the Arm, Shoulder and Hand (DASH), Foot and Ankle Ability Measure (FAAM), Knee Injury and Osteoarthritis Outcome Score (KOOS), and others [1, 3]. However, most of these instruments are narrow in scope, limited to specific outcomes or mobility constructs [1, 3]. The National Institute of Health (NIH) Patient Reported Outcomes Measurement Information System (PROMIS) was developed to deliver standardized, precise, quantitative values for individual domains of health and well-being [4], and has great potential to improve understandings of PROs in orthopaedic cases. By design, PROMIS outcome instruments report outcomes utilizing standardized T-scores. The computerized adaptive test (CAT) feature, available for many PROMIS instruments, is the most efficient method of collecting useful PROs in a multitude of musculoskeletal conditions by utilizing item response theory [1, 5, 6]. Patients respond to questions and the system is programmed to select subsequent questions based on answers of previous questions, which minimizes the burden on the patient while providing maximally useful information for clinicians [6]. The most important outcome domain in orthopaedic surgery is physical health (PH) [7]. In PROMIS, PH includes Physical Function (PF) and subdomains, such as Pediatric Mobility, Upper Extremity (UE), and Lower Extremity (LE) [8]. PROMIS PF CAT selects from a 124-item bank [6], and requires 12 or fewer questions to identify the most informative PF value [5]. While PROMIS PF CAT was not designed for any particular disease, the range of PRO values available allow it to be tailored for use in specific medical conditions.

Psychometric validation of HRQoL assessment instruments generally requires evaluation of reliability, responsiveness, and validity [9, 10]. However, the use of different terminology for the same measurement properties can complicate the consensus for validity of an assessment instrument [11]. While Sullivan established guidelines for assessing the validity of PROs assessment instruments [12], and the Consensus-based Standards for the selection of health status Measurement Instruments (COSMIN) study developed international agreement on taxonomy, terminology, and definitions of measurement properties [11], the subsequent application of these terms remains to be evaluated.

A scoping study allows us to establish uniform application of terms and definitions for assessing validity of measurement properties as applied in orthopaedic research. This approach is defined by Daudt et al*.*, as an attempt to “map the literature on a particular topic or research area and provide an opportunity to identify key concepts; gaps in the research; and types and sources of evidence to inform practice, policymaking, and research” [13]. A key feature of a scoping study is that the research aims to provide an overview of all existing literature concerning a broad topic [13, 14], whereas the purpose of a systematic review is to provide a summary of the leading existing research on a specific question [15].

HRQoL assessment instruments provide patients an opportunity to better understand their medical condition and be involved in their own care—key steps in reaching an appropriate and successful treatment plan. Given growing recognition of the importance of patients’ involvement in their own care, PROMIS is a measurement system which contains unique measures for improving patient care throughout orthopaedics. The efficacy of PROMIS has been demonstrated in many diseases including rheumatoid arthritis, chronic heart failure, and cancer [16]. However, its application throughout orthopaedic care has yet to be depicted. This scoping study sought to elucidate the extent to which orthopaedic surgery subspecialties have used and validated PROMIS measures in peer-reviewed research in order to identify its potential as an applicable and valuable tool across subspecialties in orthopaedics.

## Methods

### Approach

This study followed the methodology developed by Arksey and O’Malley [14], and further enhanced by Daudt et al*.* [13]. The Preferred Reporting Items for Systematic Reviews and Meta-Analyses extension for Scoping Reviews (PRISMA-ScR) checklist was followed for reporting the results of the study [17].

### Data source

We identified all peer-reviewed publications in the National Library of Medicine (NLM) PubMed database that utilized PROMIS measures within adult orthopaedic surgery using specific search criteria: (PROMIS) AND (orthopaedics OR orthopaedic OR orthopedics OR orthopedic). The NLM PubMed database search was conducted on January 1, 2021. This search identified both assessments of surgical patient outcomes with PROMIS and analyses of the quality of PROMIS as a measurement system within orthopaedic surgery. Pediatric publications, literature reviews, and publications that were unrelated to the care of orthopaedic patients or did not utilize PROMIS in the study were excluded from analysis as identified by individual review of publications. The full text of each publication was independently reviewed by one of two reviewers.

### Outcomes and variables collected

We then evaluated and charted each publication for study design, level of evidence, number of patients, and PROMIS domains and instrument format tested. Publications were separated by orthopaedic surgery subspecialties, including Foot and Ankle (FA), Hand and Upper Extremity (HUE), Tumor, Trauma, Adult Reconstruction (AR), Sports, Spine, and General Orthopaedics. We excluded review and editorial publications as well as pediatric orthopaedic surgery publications. Level of evidence was determined following the updated assignments provided by the Journal of Bone and Joint Surgery [18]. PROMIS domains included Global Health (Physical and Mental), PF, UE, LE, Pain Interference, Pain Intensity, Pain Behavior, Depression, Anxiety, Social Satisfaction, and Fatigue. Instrument format of PROMIS domains included CAT or short form. Study design included prospective, retrospective, cross-sectional, randomized controlled trial (RCT), and case report or series.

### Assessment of PROMIS validation studies

After initial review, we further assessed each publication to determine whether the performance of PROMIS PH CAT measurement instruments (PF, UE, and LE) use in orthopaedic surgery care was analyzed following the validation guidelines developed by Sullivan [12], with respect to reliability, responsiveness, and validity following terminology defined by COSMIN [11]. In this study, a PROMIS validation study refers to a publication that statistically analyzed a PROMIS domains’ reliability [19], responsiveness [20], or validity [21], using the following statistical tests described [22, 23]. The statistical analysis of a PROMIS domain’s validity relates to the evaluation of content validity, construct validity, or criterion validity [11]. Each validation study was assessed for any recommendations on whether the PROMIS PH CAT domains utilized (PF, UE, and LE) were accurate and useful in orthopaedic patients.

Reliability including internal consistency and inter- and intra-rater reliability, was presented by Cronbach’s alpha, kappa statistics, percentage agreement, or a correlation coefficient [11, 24].

Internal and external responsiveness was assessed using a range of statistical tests including effect size, standardized response mean, relative efficiency statistic, the response statistic, and correlation (using Spearman’s rho) [11, 20, 25]. While evaluating the minimal clinically important difference and floor and ceiling effects of assessment instruments risks spurious change and does not maintain the same statistical integrity as the prior evaluation tests, studies that calculated these values were included as psychometric tests of responsiveness, as these calculations are necessary to measure responsiveness of a given instrument [26].

Modern validity theory from the psychometric perspective requires specific contexts to be evaluated in order to assess the validity of a PROs assessment instruments [27, 28]. Therefore, the types of validity evaluated by this study looked to denote how interpretable PROMIS PH CAT scores are in various contexts of orthopaedic clinical care and research [29]. We included the three types of validity defined by COSMIN when evaluating the performance of PROMIS PH CAT: content validity, construct validity, and criterion validity [11]. Assessment of content validity uses judgements from experts in the field to give a scale of relevance for the construct or the dimensions of the construct evaluated and an average relevance is calculated [30]. Additionally, confirmatory factor analysis, a special form of structural equating modeling, analyzes specific structures and components of the construct through correlations between latent variables: mathematically inferred variables from observed variables [31]. Use of structural equating modeling to confirm specified relationships between PROMIS domains and events of interest in a disease or treatment qualified as measurement of content validity in the publications found [31, 32].

Assuming content validity, construct validity evaluates the consistency of the assessment instrument with different hypotheses [11]. For instance, evaluating construct validity can refer to the ability of PROMIS to discriminate between relevant groups or confirm relationships to known risk factors [11, 21]. Several methods for testing construct validity have been described including correlation calculations such as Pearson’s rho, multivariate analysis, confirmatory factor analysis, and covariance component analysis [33, 34]. Finally, criterion validity refers to the degree to which the assessment instrument correlates to previously validated or gold standard instruments [11], as tested by correlation coefficients [35].

## Results

### Selection of sources of evidence

**Fig. 1:**
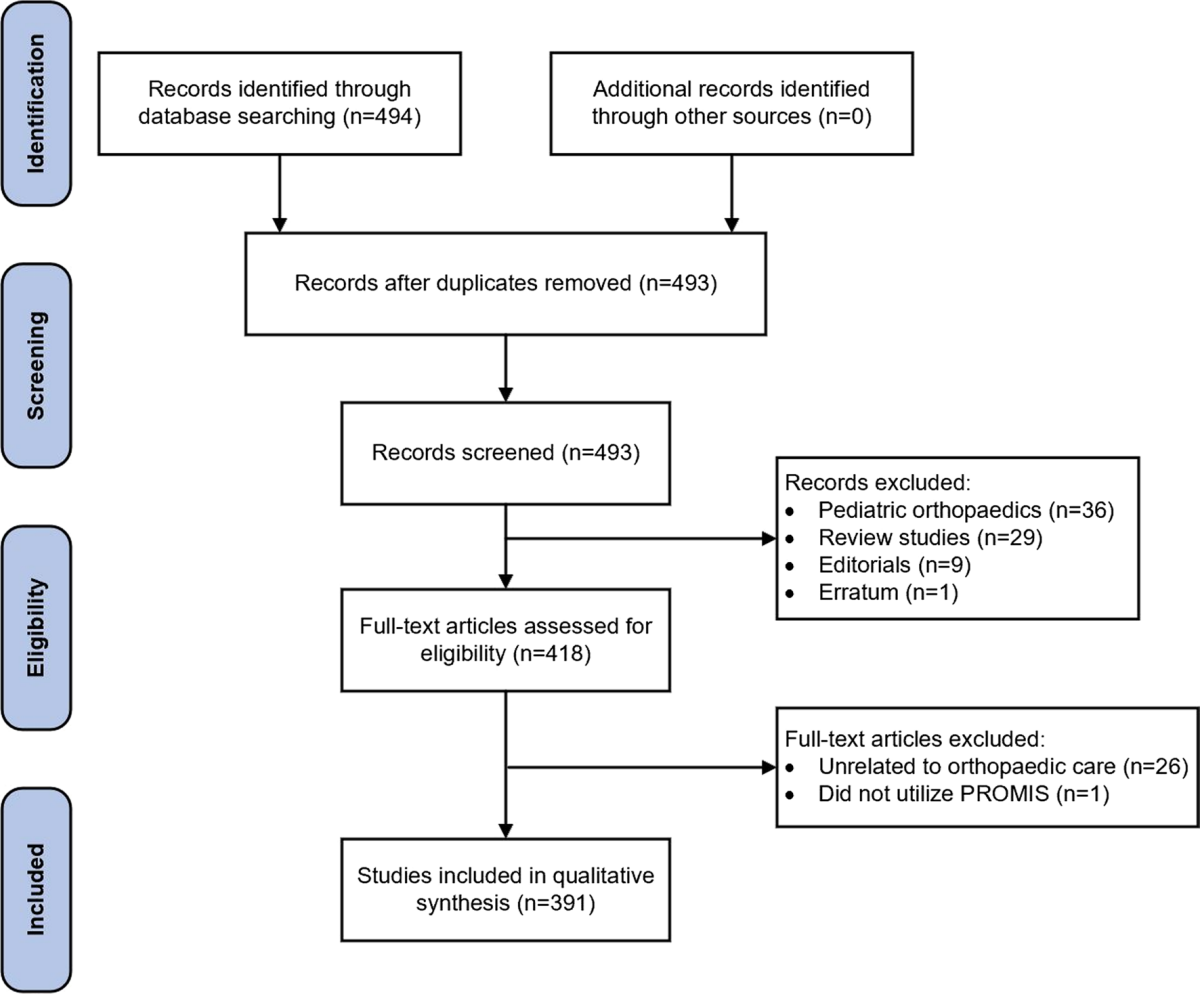
The preferred reporting items for systematic reviews of meta-analyses flow diagram for Patient-Reported Outcomes Information System (PROMIS) publications in adult orthopaedic surgery collected

The NLM PubMed database identified 493 non-duplicated publications. Individual review of the search results identified 102 publications that were not related to the primary objective of the search criteria and therefore excluded from further analysis: 36 pediatric orthopaedics publications, 29 review studies, 9 editorials, 1 published erratum, and 27 publications that were unrelated to the care of orthopaedic patients or did not utilize PROMIS in the study. The Preferred Reporting Items for Systematic Reviews of Meta-Analyses (PRISMA) diagram in Fig. 1 illustrates the sequence of review results collected in this study [36].

### All orthopaedic surgery PROMIS publications

**Fig. 2:**
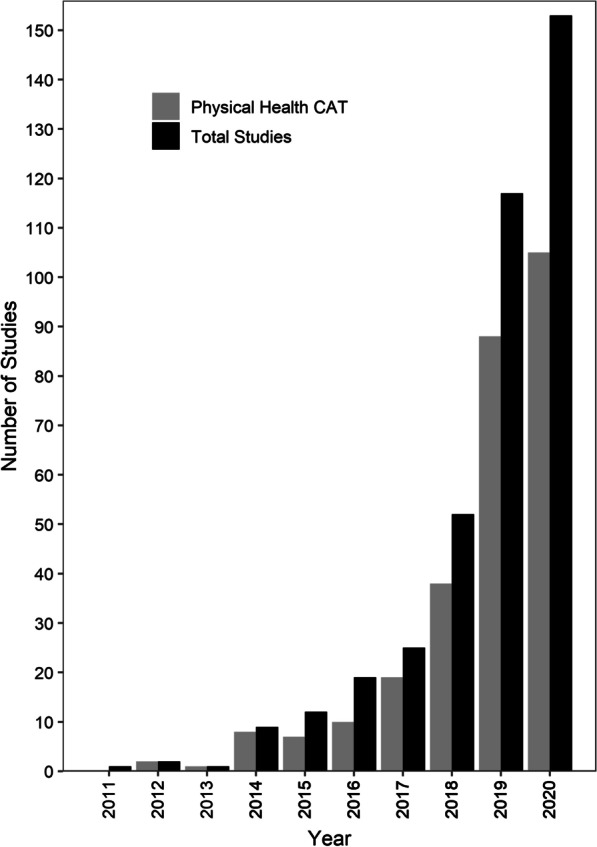
Number of all adult orthopaedic surgery Patient-Reported Outcomes Information System (PROMIS) studies and PROMIS physical health computerized adaptive test (CAT) studies (Physical Function, Upper Extremity, and Lower Extremity) published each year from 2011 through December 31, 2020

Of the total 391 publications assessed (Additional File 1), 153 (39%) were PROMIS PH CAT validation publications. From 2011 through 2020 there were increasingly more orthopaedic PROMIS studies published each year in all specialties except for Trauma (Table 1) and an increase in the number of studies investigating or utilizing PROMIS PH CAT domains in all specialties (Fig. 2). PROMIS publications most often reported HUE outcomes (26%, *n* = 100), followed by Spine (18%, *n* = 69) (Table 2). More Level I (8%, *n* = 3) and RCT (11%, *n* = 4) studies were published in the Trauma subspecialty relative to other subspecialties. UE, Pain Interference, and Depression domains were utilized the most frequently in HUE; PF and Pain Intensity domains were utilized the most in Spine. Six percent (*n* = 22) of publications were Level I; 33% (*n* = 129) were Level II; 50% (*n* = 196) were Level III; 11% (*n* = 43) were Level IV.

**Table 1:** Characteristics of all PROMIS publications by publication year

Variable	Publication year									
	2011	2012	2013	2014	2015	2016	2017	2018	2019	2020
n	1	2	1	**9**	12	19	25	52	117	153
Number of patients, median (IQR)	865 (865–865)	334.5 (311–358)	288 (288–288)	140 (126–311)	129 (49–151)	93 (31–118)	109 (74–300)	161 (91–1027)	172 (94–489)	166 (86–351)
*Subspecialty, n (%)*										
Adult reconstruction	–	–	–	–	–	–	–	9 (17)	12 (10)	23 (15)
Foot and ankle	–	2 (100)	1 (100)	3 (33)	–	3 (16)	1 (4)	2 (4)	9 (8)	22 (14)
General orthopaedics	1 (100)	–	–	–	–	–	1 (4)	5 (10)	13 (11)	11 (7)
Hand and upper extremity	–	–	–	3 (33)	7 (58)	7 (37)	8 (32)	12 (23)	28 (24)	35 (23)
Spine	–	–	–	1 (11)	1 (8)	1 (5)	4 (16)	8 (15)	24 (21)	30 (20)
Sports	–	–	–	1 (11)	–	2 (11)	4 (16)	7 (14)	12 (10)	17 (11)
Trauma	–	–	–	1 (11)	12 (17)	3 (16)	2 (8)	7 (14)	11 (9)	11 (7)
Tumor	–	–	–	–	12 (17)	3 (16)	5 (20)	2 (4)	8 (7)	4 (3)
*Publication type, n (%)*										
PROMIS validation	1 (100)	2 (100)	1 (100)	8 (89)	3 (25)	8 (42)	16 (64)	26 (50)	59 (50)	53 (35)
Patient outcomes	–	–	–	1 (11)	9 (75)	11 (58)	9 (36)	26 (50)	58 (50)	100 (65)
*Study design, n (%)*										
Case report or series	–	–	–	1 (11)	1 (8)	2 (11)	–	2 (4)	5 (4)	13 (9)
Cross sectional	–	–	–	–	3 (25)	3 (16)	3 (12)	11 (21)	11 (9)	8 (5)
Prospective cohort	1 (100)	1 (50)	1 (100)	6 (67)	3 (25)	8 (42)	12 (48)	18 (35)	36 (31)	33 (22)
Retrospective cohort	–	–	–	–	4 (33)	6 (32)	9 (36)	17 (33)	62 (53)	92 (60)
Randomized controlled trial	–	1 (50)	–	2 (22)	1 (8)	–	1 (4)	4 (8)	3 (3)	7 (5)
*Level of evidence, n (%)*										
I	–	–	–	3 (33)	1 (8)	1 (5)	1 (4)	6 (12)	4 (3)	6 (4)
II	1 (100)	1 (50)	–	3 (33)	2 (17)	8 (42)	12 (50)	18 (35)	43 (37)	40 (26)
III	–	1 (50)	1 (100)	2 (22)	5 (42)	6 (32)	10 (42)	25 (48)	56 (48)	91 (59)
IV	–	–	–	1 (11)	4 (33)	4 (21)	1 (4)	3 (6)	14 (12)	16 (11)
*PROMIS format, n (%)*										
CAT	1 (100)	2 (100)	1 (100)	9 (100)	9 (75)	14 (74)	20 (80)	42 (81)	92 (79)	111 (73)
Short form	–	–	–	–	3 (25)	2 (11)	6 (24)	12 (23)	26 (22)	47 (31)
Format unknown	–	–	–	–	–	3 (16)	–	–	2 (2)	1 (1)
*PROMIS domains, n (% of specified publication year)*										
Global physical and mental health	–	–	–	–	–	2 (11)	2 (8)	7 (14)	10 (9)	30 (20)
Physical function	1 (100)	–	1 (100)	6 (67)	7 (58)	11 (58)	16 (64)	37 (71)	83 (71)	102 (67)
Upper extremity	–	–	–	1 (11)	1 (8)	3 (16)	11 (44)	12 (23)	33 (28)	28 (18)
Lower extremity	–	2 (100)	–	2 (22)	–	–	1 (4)	–	–	–
Pain interference	–	–	1 (100)	1 (11)	7 (58)	12 (63)	14 (56)	29 (56)	65 (56)	82 (54)
Pain intensity	–	–	–	–	1 (8)	3 (16)	4 (16)	4 (8)	17 (15)	18 (12)
Pain behavior	–	–	–	1 (11)	2 (17)	1 (5)	1 (4)	1 (2)	5 (4)	2 (1)
Depression	–	–	–	–	6 (50)	6 (32)	7 (28)	16 (31)	33 (28)	51 (33)
Anxiety	–	–	–	–	3 (25)	1 (5)	7 (28)	6 (12)	13 (11)	18 (12)
Social satisfaction	–	–	–	–	1 (8)	–	3 (12)	3 (6)	6 (5)	14 (9)
Fatigue	–	–	–	–	1 (8)	–	3 (12)	3 (6)	6 (5)	11 (7)

*IQR* interquartile range, *CAT* computerized adaptive test

**Table 2:** Characteristics of all PROMIS publications by orthopaedic subspecialty

Variable	Orthopaedic subspecialty							
	Adult reconstruction	Foot and ankle	General orthopaedics	Hand and upper extremity	Spine	Sports	Trauma	Tumor
n	44	43	31	100	69	43	37	24
Number of patients, median (IQR)	186 (96–421)	148 (73–294)	435 (256–2566)	140.5 (88–351)	167 (98–421)	145 (58–272)	134 (63–198)	91 (33–138)
*Publication type, n (%)*								
PROMIS validation	18 (41)	17 (40)	12 (39)	47 (47)	40 (58)	25 (58)	11 (30)	7 (29)
Patient outcomes	26 (59)	26 (60)	19 (61)	53 (53)	29 (42)	18 (42)	26 (70)	17 (71)
*Study design, n (%)*								
Case report or series	4 (9)	3 (7)	–	8 (8)	1 (1)	2 (5)	4 (11)	2 (8)
Cross sectional	2 (5)	1 (2)	8 (26)	15 (15)	2 (3)	5 (12)	2 (5)	4 (17)
Prospective cohort	9 (20)	15 (35)	11 (36)	37 (37)	15 (22)	16 (37)	11 (30)	5 (21)
Retrospective cohort	27 (61)	24 (56)	10 (32)	36 (36)	50 (73)	17 (40)	16 (43)	13 (54)
Randomized controlled trial	2 (5)	–	2 (6)	4 (4)	1 (1)	3 (7)	4 (11)	–
*Level of evidence, n (%)*								
I	3 (7)	3 (7)	2 (6)	6 (6)	2 (3)	3 (7)	3 (8)	–
II	8 (18)	12 (28)	13 (42)	41 (41)	23 (33)	15 (35)	12 (32)	5 (22)
III	26 (59)	23 (53)	16 (52)	43 (43)	40 (58)	21 (49)	15 (41)	12 (52)
IV	7 (16)	5 (12)	–	10 (10)	4 (6)	4 (9)	7 (19)	6 (26)
*PROMIS format, n (%)*								
CAT	20 (46)	37 (86)	25 (81)	92 (92)	55 (80)	35 (81)	27 (73)	9 (38)
Short form	25 (57)	9 (21)	6 (19)	9 (9)	14 (20)	8 (19)	10 (27)	15 (63)
Format unknown	–	2 (5)	1 (3)	1 (1)	–	–	1 (3)	1 (4)
*PROMIS domains, n (% of specified subspecialty)*								
Global physical and mental health	20 (45)	6 (14)	3 (10)	5 (5)	8 (12)	4 (9)	2 (5)	3 (13)
Physical function	23 (52)	38 (88)	21 (68)	45 (43)	61 (88)	33 (77)	27 (73)	16 (67)
Upper extremity	1 (2)	–	3 (10)	61 (61)	1 (1)	10 (23)	9 (24)	4 (17)
Lower extremity	–	3 (7)	–	–	–	1 (2)	–	1 (4)
Pain interference	12 (27)	35 (81)	20 (65)	56 (56)	37 (54)	21 (49)	15 (41)	15 (63)
Pain intensity	2 (5)	8 (19)	3 (10)	5 (5)	15 (22)	3 (7)	2 (5)	9 (38)
Pain behavior	1 (2)	2 (5)	1 (3)	2 (2)	2 (3)	2 (5)	1 (3)	2 (8)
Depression	7 (16)	19 (44)	17 (55)	35 (35)	13 (19)	15 (35)	2 (5)	11 (46)
Anxiety	3 (7)	2 (5)	13 (42)	9 (9)	8 (12)	4 (9)	–	9 (38)
Social satisfaction	1 (2)	1 (2)	8 (26)	2 (2)	7 (10)	3 (7)	1 (3)	4 (17)
Fatigue	2 (5)	1 (2)	7 (23)	2 (2)	5 (7)	3 (7)	–	4 (17)

*IQR* interquartile range, *CAT* computerized adaptive test

PF (I: 50%, *n* = 11; II: 73%, *n* = 94; III: 68%, *n* = 133; IV: 58%, *n* = 25) and then Pain Interference (I: 36%, *n* = 8; II: 63%, *n* = 81; III: 51%, *n* = 99; IV: 53%, *n* = 23) were utilized the most within each level of evidence degree, and within each orthopaedic subspecialty with the exception of General Orthopaedics (Table 3).

**Table 3:** Characteristics of all PROMIS publications by level of evidence

Variable	Level of evidence			
	I	II	III	IV
n	22	129	196	43
Number of patients, median (IQR)	130 (56–207)	183 (100–472)	172 (100–373)	27 (16–66)
*Year, n (%)*				
2011	–	1 (1)	–	–
2012	–	1 (1)	1 (1)	–
2013	–	1 (1)	–	–
2014	3 (16)	3 (3)	2 (1)	1 (3)
2015	1 (5)	2 (2)	5 (3)	4 (10)
2016	1 (5)	8 (7)	6 (4)	4 (10)
2017	1 (5)	12 (10)	10 (6)	1 (3)
2018	6 (32)	18 (15)	25 (15)	3 (8)
2019	4 (21)	43 (36)	56 (32)	14 (35)
2020	6 (27)	40 (31)	91 (46)	16 (37)
*Subspecialty, n (%)*				
Adult reconstruction	3 (14)	8 (6)	26 (13)	7 (16)
Foot and ankle	3 (14)	12 (9)	23 (12)	5 (12)
General orthopaedics	2 (9)	13 (10)	16 (8)	–
Hand and upper extremity	6 (27)	41 (32)	43 (22)	10 (23)
Spine	2 (9)	23 (18)	40 (20)	4 (9)
Sports	3 (14)	15 (12)	21 (11)	4 (9)
Trauma	3 (14)	12 (9)	15 (8)	7 (16)
Tumor	–	5 (4)	12 (6)	6 (14)
*Publication type, n (%)*				
PROMIS validation	7 (32)	89 (69)	76 (39)	4 (9)
Patient outcomes	15 (68)	40 (31)	120 (61)	39 (91)
*Study design, n (%)*				
Case report or series	–	–	–	24 (56)
Cross sectional	–	4 (3)	33 (17)	2 (5)
Prospective cohort	7 (32)	105 (81)	5 (3)	1 (2)
Retrospective cohort	1 (4)	19 (15)	157 (80)	16 (37)
Randomized controlled trial	14 (64)	1 (1)	1 (1)	–
*PROMIS format, n (%)*				
CAT	15 (68)	112 (87)	148 (76)	25 (58)
Short form	8 (36)	19 (15)	53 (27)	15 (35)
Format unknown	–	1 (1)	2 (1)	3 (7)
*PROMIS domains, n (% of specified level of evidence)*				
Global physical and mental health	3 (14)	14 (11)	30 (15)	4 (9)
Physical function	11 (50)	94 (73)	133 (68)	25 (58)
Upper extremity	5 (23)	35 (27)	43 (22)	5 (12)
Lower extremity	1 (5)	2 (2)	2 (1)	–
Pain interference	8 (36)	81 (63)	99 (51)	23 (53)
Pain intensity	3 (14)	16 (12)	23 (12)	5 (12)
Pain behavior	1 (5)	5 (4)	3 (2)	4 (9)
Depression	3 (14)	39 (30)	65 (33)	12 (28)
Anxiety	1 (5)	18 (14)	23 (12)	6 (14)
Social satisfaction	3 (14)	13 (10)	9 (5)	2 (5)
Fatigue	1 (5)	11 (9)	8 (4)	4 (9)

*IQR* interquartile range, *CAT* computerized adaptive test

### Orthopaedic surgery physical health PROMIS validation publications

Ninety-five percent (*n* = 146) of all orthopaedic surgery PROMIS PH CAT validation publications determined that the instruments were responsive, reliable, and valid. Two studies in AR (18%), two in HUE (5%), two in Sports (8%), and one in Trauma (10%) did not find PROMIS PH CAT instruments to be valid instrument within their respective field. Specifically, these studies found problems with PROMIS PH CAT criterion validity and responsiveness.

Eighty-five percent (*n* = 130) of all orthopaedic surgery PROMIS PH CAT validation publications analyzed PF, 30% (*n* = 46) analyzed UE, and 3% (*n* = 4) analyzed LE. More PROMIS PH CAT validation publications were performed in 2019 (35%, *n* = 53) than any other year (Table 4). PROMIS PH CAT validation publications most often reported HUE outcomes (26%, *n* = 40), followed by Spine (23%, *n* = 35) and Sports (16%, *n* = 24) (Table 5).

**Table 4:** Characteristics of publications validating physical health CAT PROMIS domains by publication year (includes physical function, upper extremity, lower extremity)

Variable	Publication year								
	2012	2013	2014	2015	2016	2017	2018	2019	2020
n	2	1	8	1	7	15	23	53	43
Number of patients, median (IQR)	334.5 (311–358)	288 (288–288)	147 (132–343)	187 (187–187)	108 (82–204)	107 (84–726)	231 (94–1969)	173 (100–748)	166 (115–259)
*Subspecialty, n (%)*									
Adult reconstruction	–	–	–	–	–	–	4 (17)	1 (2)	6 (14)
Foot and ankle	2 (100)	1 (100)	3 (38)	–	–	1 (7)	1 (4)	5 (9)	4 (9)
General orthopaedics	–	–	–	–	–	–	4 (17)	5 (9)	1 (2)
Hand and upper extremity	–	–	2 (25)	1 (100)	2 (29)	6 (40)	4 (17)	13 (25)	12 (28)
Spine	–	–	1 (13)	–	1 (14)	2 (13)	5 (22)	15 (28)	11 (26)
Sports	–	–	1 (13)	–	1 (14)	3 (20)	3 (13)	8 (15)	8 (19)
Trauma	–	–	1 (13)	–	2 (29)	1 (7)	2 (9)	4 (8)	–
Tumor	–	–	–	–	1 (14)	2 (13)	–	2 (4)	1 (2)
*Study design, n (%)*									
Case report or series	–	–	–	–	–	–	1 (4)	–	1 (2)
Cross sectional	–	–	–	–	2 (29)	2 (13)	5 (22)	6 (11)	2 (5)
Prospective cohort	1 (50)	1 (100)	6 (75)	–	4 (57)	10 (67)	12 (52)	29 (55)	10 (23)
Retrospective cohort	1 (50)	–	2 (25)	1 (100)	1 (14)	3 (20)	5 (22)	18 (34)	30 (70)
*Level of evidence, n (%)*									
I	–	–	3 (38)	–	–	–	2 (9)	2 (4)	–
II	1 (50)	1 (100)	3 (38)	–	4 (57)	10 (67)	12 (52)	32 (60)	14 (33)
III	1 (50)	–	2 (25)	1 (100)	3 (43)	5 (33)	8 (35)	18 (34)	27 (63)
IV	–	–	–	–	–	–	1 (4)	1 (2)	2 (5)
*PROMIS domains, n (% of specified publication year)*									
Physical function	–	1 (100)	6 (75)	1 (100)	6 (86)	13 (87)	21 (91)	50 (94)	32 (74)
Upper extremity	–	–	1 (13)	–	2 (29)	8 (53)	5 (22)	15 (28)	15 (35)
Lower extremity	2 (100)	–	2 (25)	–	–	–	–	–	–
*Validation method tested, n (%)*									
Reliability	2 (100)	1 (100)	7 (88)	1 (100)	3 (43)	3 (20)	1 (4)	8 (15)	3 (7)
Responsiveness	2 (100)	1 (100)	8 (100)	1 (100)	2 (29)	12 (80)	13 (57)	41 (77)	30 (70)
Validity									
1 validity criteria	1 (50)	–	2 (25)	–	6 (86)	7 (47)	15 (65)	28 (53)	32 (74)
> 1 validity criterion	1 (50)	1 (100)	6 (75)	1 (100)	1 (14)	3 (20)	2 (9)	9 (17)	3 (7)

*IQR* interquartile range, *CAT* computerized adaptive test

**Table 5:** Characteristics of publications validating physical health CAT PROMIS domains by orthopaedic subspecialty (includes physical function, upper extremity, lower extremity)

Variable	Orthopaedic subspecialty							
	Adult reconstruction	Foot and ankle	General orthopaedics	Hand and upper extremity	Spine	Sports	Trauma	Tumor
n	11	17	10	40	35	24	10	6
Number of patients, median (IQR)	172 (105–762)	288 (126–441)	2566 (566–12,353)	169.5 (100–782)	158 (120–360)	138.5 (76–255)	163.5 (123–208)	100 (98–115)
*Study design, n (%)*								
Case report or series	1 (9)	–	–	–	–	1 (4)	–	–
Cross sectional	1 (9)	–	3 (30)	5 (13)	2 (6)	3 (13)	2 (20)	1 (17)
Prospective cohort	4 (36)	10 (59)	5 (50)	21 (53)	12 (34)	13 (54)	5 (50)	3 (50)
Retrospective cohort	5 (46)	7 (41)	2 (20)	14 (35)	21 (60)	7 (29)	3 (30)	2 (33)
*Level of evidence, n (%)*								
I	1 (9)	3 (18)	–	2 (5)	1 (3)	–	–	–
II	3 (27)	7 (41)	6 (60)	22 (55)	17 (49)	13 (54)	6 (60)	3 (50)
III	6 (55)	7 (41)	4 (40)	16 (40)	16 (46)	9 (38)	4 (40)	3 (50)
IV	1 (9)	–	–	–	1 (3)	2 (8)	–	–
*PROMIS domains, n (% of specified subspecialty)*								
Physical function	11 (100.0)	15 (88)	9 (90)	22 (55)	35 (100)	22 (92)	10 (100)	6 (100)
Upper extremity	1 (9)	–	2 (20)	32 (80)	1 (3)	6 (25)	3 (30)	1 (17)
Lower extremity	–	3 (18)	–	–	–	1 (42)	–	–
*Validation method tested, n (%)*								
Reliability	–	6 (35)	1 (10)	12 (30)	3 (9)	1 (4)	2 (20)	4 (67)
Responsiveness	7 (64)	15 (88)	2 (20)	33 (83)	21 (60)	19 (79)	7 (70)	6 (100)
Validity								
1 validity criteria	8 (73)	7 (41)	8 (80)	21 (53)	20 (57)	16 (67)	8 (80)	3 (50)
> 1 validity criterion	–	4 (24)	–	9 (23)	9 (26)	2 (8)	1 (10)	2 (33)

*IQR* interquartile range, *CAT* computerized adaptive test

Reliability was the least-often analyzed component of PROMIS PH CAT performance throughout each subspecialty (range, 4–67%), as compared to responsiveness or validity. Reliability was analyzed most frequently in HUE validation studies (*n* = 12), followed by FA validation studies (*n* = 6). Responsiveness was analyzed most frequently in HUE validation studies (*n* = 33), followed by Spine (*n* = 21) and Sports (*n* = 19) studies. At least one form of validity (criterion, content, or construct) was analyzed in over 65% of all subspecialties and analyzed in over 80% of General Orthopaedics, Spine, Trauma, and Tumor validation studies. More than one form of validity was analyzed in over 20% of FA, HUE, Spine, and Tumor validation studies. Five percent (*n* = 7) of validation publications were Level I studies; 50% (*n* = 77) were Level II studies; 42% (*n* = 65) were Level III; 3% (*n* = 4) were Level IV (Table 6). The majority of Level I studies were performed in FA (43%, *n* = 3), Level II studies in HUE (29%, *n* = 22), and Level III studies in both HUE and Spine (25%, *n* = 16).

**Table 6:** Characteristics of publications validating physical health CAT PROMIS domains by level of evidence (includes physical function, upper extremity, lower extremity)

Variable	Level of evidence			
	I	II	III	IV
n	7	77	65	4
Number of patients, median (IQR)	983 (219–2507)	157 (100–734)	166 (108–290)	85.5 (67–135)
*Year, n (%)*				
2012	–	1 (1)	1 (2)	–
2013	–	1 (1)	–	–
2014	3 (43)	3 (4)	2 (3)	–
2015	–	–	1 (2)	–
2016	–	4 (5)	3 (5)	–
2017	–	10 (13)	5 (8)	–
2018	2 (29)	12 (16)	8 (12)	1 (25)
2019	2 (29)	32 (42)	18 (28)	1 (25)
2020	–	14 (18)	27 (42)	2 (50)
*Subspecialty, n (%)*				
Adult reconstruction	1 (14)	3 (4)	6 (9)	1 (25)
Foot and ankle	3 (43)	7 (9)	7 (11)	–
General orthopaedics	–	6 (8)	4 (6)	–
Hand and upper extremity	2 (29)	22 (29)	16 (25)	–
Spine	1 (14)	17 (22)	16 (25)	1 (25)
Sports	–	13 (17)	9 (14)	2 (50)
Trauma	–	6 (8)	4 (6)	–
Tumor	–	3 (4)	3 (5)	–
*Study design, n (%)*				
Case report or series	–	–	–	2 (50)
Cross sectional	–	1 (1)	16 (25)	–
Prospective cohort	6 (86)	65 (84)	2 (3)	–
Retrospective cohort	1 (14)	11 (14)	47 (72)	2 (50)
*PROMIS domains, n (% of specified level of evidence)*				
Physical function	6 (86)	66 (86)	54 (83)	4 (100)
Upper extremity	2 (29)	27 (35)	17 (26)	–
Lower extremity	1 (14)	2 (3)	1 (2)	–
*Validation method tested, n (%)*				
Reliability	4 (57)	14 (18)	11 (17)	–
Responsiveness	7 (100)	57 (74)	43 (66)	3 (75)
Validity				
1 validity criteria	–	42 (55)	46 (71)	3 (75)
> 1 validity criterion	4 (57)	17 (22)	6 (9)	–

*IQR* interquartile range, *CAT* computerized adaptive test

PROMIS PF CAT specifically was validated in 130 studies, 50% of which were Level II studies. Since 2011, PROMIS PF CAT was analyzed for reliability 29 times, responsiveness 110 times, and at least one form of validity 118 times.

## Discussion

The increased utilization of PROMIS measurement instruments across all types of orthopaedic surgery has enabled surgeons to gain a deeper understanding of patients’ physical and mental health while engaging patients more directly in their care. Compared to legacy measurement instruments (ASES, DASH, FAAM, KOOS) which are generally narrow in scope and can incur patient and administrative burden [1, 3], PROMIS CATs have the capacity to be tuned to orthopaedic diseases and improve patients’ experiences in orthopaedic surgery clinics [37]. These tests are enabling surgeons to interpret the patient’s HRQoL before and after treatment [3]. Additionally, understanding the degree and impact of a patient’s pain provides surgeons with a metric for tailoring treatment to each patient’s specific goals and needs, whether that be surgical or medical management [38, 39]. This scoping study demonstrates that in addition to becoming a more frequent subject of analysis, the PROMIS PH CAT domains (PF, UE, and LE) have repeatedly been shown to be reliable, responsive, and interpretable instruments when utilized in most contexts of orthopaedic surgery.

This scoping study determined that from January 1, 2011, through December 31, 2020, the PROMIS PH CAT was found to be interpretable as analyzed by at least one type of validity in various contexts throughout all orthopaedic surgery subspecialties in a total of 146 studies. In particular, PROMIS PF CAT was interpretable in 130 studies, 50% of which were Level II studies. Specific PROMIS PH CAT subdomains were first proposed in 2011 by Hung et al*.* [8], and have since been tested for reliability 29 times, responsiveness 110 times, and at least one form of validity 118 times. The extensive analysis of PROMIS PH CAT validity demonstrates the potential of PROMIS to assess PH in orthopaedic surgery patients. More importantly, this establishes an instrument that should effectively depict the patient’s perception of physical function status. As a widely interpretable outcome assessment instrument, PROMIS PH CAT may benefit patient care and advance orthopaedic outcomes research.

While PROMIS CAT is being shown to be interpretable more frequently and in more contexts, several limitations remain. Integrating these measurement instruments into electronic medical records remains a substantial obstacle, predominately due to financial, logistic, and technological barriers [40]. However, large-scale clinical implementation is possible and has valuable potential for improved patient care and experience [41]. Furthermore, while the short form format of PROMIS allows it to be administered as a physical test, the CAT format requires extra technology. The potential benefits outlined above may outweigh these costs in many settings. Additionally, CAT has been shown to have an improved ability to distinguish between two patients with similar health status [42], which can provide valuable insight when distinguishing between small details that can improve capabilities to diagnose and provide care.

### Limitations

We note several limitations of this analysis. The evaluation of each publication was performed by two reviewers, which risked reporting bias of selective inclusion of research findings. However, the studies analyzed had clear descriptions of collection variables and followed the terminology and guidelines created for studies validating assessment instruments [11, 12], which contributed to more reliable evaluation of publications. Utilization of specific statistical methods in evaluation of instrument validation reduced potential disagreement of publication type and analyses performed. Additionally, publications were not evaluated on quality of the results; recommendations for PROMIS instruments from validation studies were taken directly from the publication, following common methodology of scoping studies [13, 43].

Our scoping study solely searched the NLM PubMed database, which risked evidence selection bias due to the potential for missed studies published in other databases. However, the relatively high number of 391 publications demonstrated sufficient evidence of PROMIS usage in orthopaedic surgery. At the time of the search, the orthopaedic surgery Tumor subspecialty had only six PH CAT validation publications, which may be an area of further exploration. Finally, given the nature of a scoping study, the results can only be as good as the publications evaluated. Therefore, each publication was evaluated for number of patients studied and publication level of evidence, and validation was evaluated based on statistical methods. Stratification of the publications based on these variables allows readers to observe these differences and make their own inferences. Regardless of these limitations, our scoping study provides an exhaustive overview of the existing literature on the usage of PROMIS in orthopaedic surgery [13].

## Conclusions

PROMIS utilization within orthopaedics as a whole has significantly increased within the past decade, particularly within PROMIS CAT domains. The existing literature reviewed in this scoping study demonstrates that PROMIS physical health CAT domains (PF, UE, and LE) are reliable, responsive, and interpretable in most contexts of patient care throughout all orthopaedic surgery subspecialties. PROMIS enables orthopaedic surgeons to gain a deeper understanding of a patient’s physical and mental health directly from the patient, facilitating the potential to improve shared decision-making and quality of care. With numerous validation analyses of PROMIS PH CAT domains and the increasing utilization of PROMIS instruments, this study demonstrates that PROMIS PH CAT measurement instruments have much success in various contexts of orthopaedic clinical care and research. Clinicians and researchers should consider the use of PROMIS instruments within each context specifically, but in many instances, PROMIS PH CAT measures may work well in orthopaedic applications. While challenges of integrating these measurement instruments into electronic medical records exist, large-scale clinical implementation is possible and has valuable potential for improved patient care and experience; this implementation process should be an area of further research and a future healthcare objective.

## Supplementary Information

**Additional file 1: Supplementary Materials.** All manuscripts evaluated.

## Data Availability

The datasets used and analyzed during the current study are available from the corresponding author on reasonable request.

## Abbreviations

PRO: Patient reported outcome
HRQoL: Health-related quality of life
ASES: American Shoulder and Elbow Surgeons
DASH: Disabilities of the Arm, Shoulder and Hand
FAAM: Foot and Ankle Ability Measure
KOOS: Knee Injury and Osteoarthritis Outcome Score
NIH: National Institute of Health
PROMIS: Patient Reported Outcomes Measurement Information System
CAT: Computerized adaptive test
PH: Physical health
PF: Physical Function
UE: Upper Extremity
LE: Lower Extremity
COSMIN: Consensus-based Standards for the selection of health status Measurement Instruments
PRISMA-ScR: Preferred Reporting Items for Systematic Reviews and Meta-Analyses extension for Scoping Reviews
FA: Foot and Ankle
HUE: Hand and Upper Extremity
AR: Adult Reconstruction
RCT: Randomized controlled trial
