# Supplementary material for: The promise of computer adaptive testing in collection of orthopaedic outcomes: an evaluation of PROMIS utilization
Source: J Patient Rep Outcomes. 2022 Jan 4;6:2. doi: 10.1186/s41687-021-00407-w (PMC8727662; doi:10.1186/s41687-021-00407-w)
Supplement: Supplementary file 1 — Additional file 1: Supplementary Materials. All manuscripts evaluated. [file 41687_2021_407_MOESM1_ESM.docx]

**Supplemental Material.** All Manuscripts Evaluated

1. Hung M, Clegg DO, Greene T, Saltzman CL. Evaluation of the PROMIS physical function item bank in orthopaedic patients. *J Orthop Res*. 2011;29(6):947-953. doi:10.1002/jor.21308

2. Hung M, Clegg DO, Greene T, Weir C, Saltzman CL. A lower extremity physical function computerized adaptive testing instrument for orthopaedic patients. *Foot Ankle Int*. 2012;33(4):326-335. doi:10.3113/FAI.2012.0326

3. Hung M, Nickisch F, Beals TC, Greene T, Clegg DO, Saltzman CL. New paradigm for patient-reported outcomes assessment in foot & ankle research: computerized adaptive testing. *Foot Ankle Int*. 2012;33(8):621-626. doi:10.3113/FAI.2012.0621

4. Hung M, Baumhauer JF, Latt LD, et al. Validation of PROMIS ® Physical Function computerized adaptive tests for orthopaedic foot and ankle outcome research. *Clin Orthop Relat Res*. 2013;471(11):3466-3474. doi:10.1007/s11999-013-3097-1

5. Cheesborough JE, Souza JM, Dumanian GA, Bueno RA Jr. Targeted muscle reinnervation in the initial management of traumatic upper extremity amputation injury. *Hand (N Y)*. 2014;9(2):253-257. doi:10.1007/s11552-014-9602-5

6. Döring AC, Nota SP, Hageman MG, Ring DC. Measurement of upper extremity disability using the Patient-Reported Outcomes Measurement Information System. *J Hand Surg Am*. 2014;39(6):1160-1165. doi:10.1016/j.jhsa.2014.03.013

7. Hung M, Franklin JD, Hon SD, Cheng C, Conrad J, Saltzman CL. Time for a paradigm shift with computerized adaptive testing of general physical function outcomes measurements. *Foot Ankle Int*. 2014;35(1):1-7. doi:10.1177/1071100713507905

8. Hung M, Baumhauer JF, Brodsky JW, et al. Psychometric Comparison of the PROMIS Physical Function CAT With the FAAM and FFI for Measuring Patient-Reported Outcomes. *Foot Ankle Int*. 2014;35(6):592-599. doi:10.1177/1071100714528492

9. Hung M, Stuart AR, Higgins TF, Saltzman CL, Kubiak EN. Computerized Adaptive Testing Using the PROMIS Physical Function Item Bank Reduces Test Burden With Less Ceiling Effects Compared With the Short Musculoskeletal Function Assessment in Orthopaedic Trauma Patients. *J Orthop Trauma*. 2014;28(8):439-443. doi:10.1097/BOT.0000000000000059

10. Hung M, Hon SD, Franklin JD, et al. Psychometric properties of the PROMIS physical function item bank in patients with spinal disorders. *Spine (Phila Pa 1976)*. 2014;39(2):158-163. doi:10.1097/BRS.0000000000000097

11. Hung M, Hon SD, Cheng C, et al. Psychometric Evaluation of the Lower Extremity Computerized Adaptive Test, the Modified Harris Hip Score, and the Hip Outcome Score. *Orthop J Sports Med*. 2014;2(12):2325967114562191. Published 2014 Dec 19. doi:10.1177/2325967114562191

12. Hunt KJ, Alexander I, Baumhauer J, et al. The Orthopaedic Foot and Ankle Outcomes Research (OFAR) network: feasibility of a multicenter network for patient outcomes assessment in foot and ankle. *Foot Ankle Int*. 2014;35(9):847-854. doi:10.1177/1071100714544157

13. Tyser AR, Beckmann J, Franklin JD, et al. Evaluation of the PROMIS physical function computer adaptive test in the upper extremity. *J Hand Surg Am*. 2014;39(10):2047-2051.e4. doi:10.1016/j.jhsa.2014.06.130

14. Beckmann JT, Hung M, Bounsanga J, Wylie JD, Granger EK, Tashjian RZ. Psychometric evaluation of the PROMIS Physical Function Computerized Adaptive Test in comparison to the American Shoulder and Elbow Surgeons score and Simple Shoulder Test in patients with rotator cuff disease. *J Shoulder Elbow Surg*. 2015;24(12):1961-1967. doi:10.1016/j.jse.2015.06.025

15. Colman MW, Karim SM, Lozano-Calderon SA, et al. Quality of life after en bloc resection of tumors in the mobile spine. *Spine J*. 2015;15(8):1728-1737. doi:10.1016/j.spinee.2015.03.042

16. Kortlever JT, Janssen SJ, van Berckel MM, Ring D, Vranceanu AM. What Is the Most Useful Questionnaire for Measurement of Coping Strategies in Response to Nociception?. *Clin Orthop Relat Res*. 2015;473(11):3511-3518. doi:10.1007/s11999-015-4419-2

17. Hung M, Stuart A, Cheng C, et al. Predicting the DRAM mZDI using the PROMIS anxiety and depression. *Spine (Phila Pa 1976)*. 2015;40(3):179-183. doi:10.1097/BRS.0000000000000706

18. Janssen SJ, Ter Meulen DP, Nota SP, Hageman MG, Ring D. Does verbal and nonverbal communication of pain correlate with disability?. *Psychosomatics*. 2015;56(4):338-344. doi:10.1016/j.psym.2014.05.009

19. Jayakumar P, Overbeek CL, Ring DC. Relationship of age on enjoyment of physical activity in upper extremity illness. *Hand (N Y)*. 2015;10(4):767-772. doi:10.1007/s11552-015-9754-y

20. Mellema JJ, O'Connor CM, Overbeek CL, Hageman MG, Ring D. The effect of feedback regarding coping strategies and illness behavior on hand surgery patient satisfaction and communication: a randomized controlled trial. *Hand (N Y)*. 2015;10(3):503-511. doi:10.1007/s11552-015-9742-2

21. Menendez ME, Eberlin KR, Mudgal CS, Ring D. Language barriers in Hispanic patients: relation to upper-extremity disability. *Hand (N Y)*. 2015;10(2):279-284. doi:10.1007/s11552-014-9697-8

22. Morgan JH, Kallen MA, Okike K, Lee OC, Vrahas MS. PROMIS Physical Function Computer Adaptive Test Compared With Other Upper Extremity Outcome Measures in the Evaluation of Proximal Humerus Fractures in Patients Older Than 60 Years. *J Orthop Trauma*. 2015;29(6):257-263. doi:10.1097/BOT.0000000000000280

23. Okike K, Lee OC, Makanji H, Morgan JH, Harris MB, Vrahas MS. Comparison of locked plate fixation and nonoperative management for displaced proximal humerus fractures in elderly patients. *Am J Orthop (Belle Mead NJ)*. 2015;44(4):E106-E112.

24. Stuart AR, Higgins TF, Hung M, et al. Reliability in Measuring Preinjury Physical Function in Orthopaedic Trauma. *J Orthop Trauma*. 2015;29(12):527-532. doi:10.1097/BOT.0000000000000392

25. Tosi LL, Oetgen ME, Floor MK, et al. Initial report of the osteogenesis imperfecta adult natural history initiative. *Orphanet J Rare Dis*. 2015;10:146. Published 2015 Nov 14. doi:10.1186/s13023-015-0362-2

26. Beckmann JT, Hung M, Voss MW, Crum AB, Bounsanga J, Tyser AR. Evaluation of the Patient-Reported Outcomes Measurement Information System Upper Extremity Computer Adaptive Test. *J Hand Surg Am*. 2016;41(7):739-744.e4. doi:10.1016/j.jhsa.2016.04.025

27. Bozzio AE, Johnson CR, Mauffrey C. Short-term results of percutaneous treatment of acetabular fractures: functional outcomes, radiographic assessment and complications. *Int Orthop*. 2016;40(8):1703-1708. doi:10.1007/s00264-015-2987-0

28. Fuchs DJ, Ho BS, LaBelle MW, Kelikian AS. Effect of Arthroscopic Evaluation of Acute Ankle Fractures on PROMIS Intermediate-Term Functional Outcomes. *Foot Ankle Int*. 2016;37(1):51-57. doi:10.1177/1071100715597657

29. Gelhorn HL, Tong S, McQuarrie K, et al. Patient-reported Symptoms of Tenosynovial Giant Cell Tumors. *Clin Ther*. 2016;38(4):778-793. doi:10.1016/j.clinthera.2016.03.008

30. Hermanussen HH, Menendez ME, Chen NC, Ring D, Vranceanu AM. Predictors of Upper-Extremity Physical Function in Older Adults. *Arch Bone Jt Surg*. 2016;4(4):359-365.

31. Ho B, Houck JR, Flemister AS, et al. Preoperative PROMIS Scores Predict Postoperative Success in Foot and Ankle Patients. *Foot Ankle Int*. 2016;37(9):911-918. doi:10.1177/1071100716665113

32. Janssen SJ, Paulino Pereira NR, Raskin KA, et al. A comparison of questionnaires for assessing physical function in patients with lower extremity bone metastases. *J Surg Oncol*. 2016;114(6):691-696. doi:10.1002/jso.24400

33. Kim CY, Wiznia DH, Averbukh L, et al. PROMIS Computer Adaptive Tests Compared With Time to Brake in Patients With Complex Lower Extremity Trauma. *J Orthop Trauma*. 2016;30(11):592-596. doi:10.1097/BOT.0000000000000645

34. Lynch AD, Dodds NE, Yu L, Pilkonis PA, Irrgang JJ. Individuals with knee impairments identify items in need of clarification in the Patient Reported Outcomes Measurement Information System (PROMIS®) pain interference and physical function item banks - a qualitative study. *Health Qual Life Outcomes*. 2016;14:77. Published 2016 May 11. doi:10.1186/s12955-016-0478-7

35. Moradi A, Menendez ME, Kachooei AR, Isakov A, Ring D. Update of the Quick DASH Questionnaire to Account for Modern Technology. *Hand (N Y)*. 2016;11(4):403-409. doi:10.1177/1558944715628006

36. Nota SP, Spit SA, Oosterhoff TC, Hageman MG, Ring DC, Vranceanu AM. Is Social Support Associated With Upper Extremity Disability?. *Clin Orthop Relat Res*. 2016;474(8):1830-1836. doi:10.1007/s11999-016-4892-2

37. Oak SR, Strnad GJ, Bena J, et al. Responsiveness Comparison of the EQ-5D, PROMIS Global Health, and VR-12 Questionnaires in Knee Arthroscopy. *Orthop J Sports Med*. 2016;4(12):2325967116674714. Published 2016 Dec 17. doi:10.1177/2325967116674714

38. O'Connor C, Braun Y, Nota SP, Baloda T, Ring D. The Association of Complementary Health Approaches With Mood and Coping Strategies Among Orthopedic Patients. *Hand (N Y)*. 2016;11(3):295-302. doi:10.1177/1558944715620798

39. Papuga MO, Mesfin A, Molinari R, Rubery PT. Correlation of PROMIS Physical Function and Pain CAT Instruments With Oswestry Disability Index and Neck Disability Index in Spine Patients. *Spine (Phila Pa 1976)*. 2016;41(14):1153-1159. doi:10.1097/BRS.0000000000001518

40. Peters RM, Menendez ME, Mellema JJ, Ring D, Vranceanu AM. Sleep Disturbance and Upper-Extremity Disability. *Arch Bone Jt Surg*. 2016;4(1):35-40.

41. Phukan R, Herzog T, Boland PJ, et al. How Does the Level of Sacral Resection for Primary Malignant Bone Tumors Affect Physical and Mental Health, Pain, Mobility, Incontinence, and Sexual Function?. *Clin Orthop Relat Res*. 2016;474(3):687-696. doi:10.1007/s11999-015-4361-3

42. Quispe JC, Herbert B, Chadayammuri VP, et al. Transarticular plating for acute posterior sternoclavicular joint dislocations: a valid treatment option?. *Int Orthop*. 2016;40(7):1503-1508. doi:10.1007/s00264-015-2952-y

43. Souza JM, Purnell CA, Cheesborough JE, Kelikian AS, Dumanian GA. Treatment of Foot and Ankle Neuroma Pain With Processed Nerve Allografts. *Foot Ankle Int*. 2016;37(10):1098-1105. doi:10.1177/1071100716655348

44. van Leeuwen WF, van der Vliet QM, Janssen SJ, Heng M, Ring D, Vranceanu AM. Does perceived injustice correlate with pain intensity and disability in orthopaedic trauma patients?. *Injury*. 2016;47(6):1212-1216. doi:10.1016/j.injury.2016.02.018

45. Anthony CA, Glass NA, Hancock K, Bollier M, Wolf BR, Hettrich CM. Performance of PROMIS Instruments in Patients With Shoulder Instability. *Am J Sports Med*. 2017;45(2):449-453. doi:10.1177/0363546516668304

46. Anthony CA, Glass N, Hancock K, Bollier M, Hettrich CM, Wolf BR. Preoperative Performance of the Patient-Reported Outcomes Measurement Information System in Patients With Rotator Cuff Pathology. *Arthroscopy*. 2017;33(10):1770-1774.e1. doi:10.1016/j.arthro.2017.04.018

47. Beleckas CM, Padovano A, Guattery J, Chamberlain AM, Keener JD, Calfee RP. Performance of Patient-Reported Outcomes Measurement Information System (PROMIS) Upper Extremity (UE) Versus Physical Function (PF) Computer Adaptive Tests (CATs) in Upper Extremity Clinics. *J Hand Surg Am*. 2017;42(11):867-874. doi:10.1016/j.jhsa.2017.06.012

48. Braun Y, Mellema JJ, Peters RM, Curley S, Burchill G, Ring D. The relationship between therapist-rated function and patient-reported outcome measures. *J Hand Ther*. 2017;30(4):516-521. doi:10.1016/j.jht.2016.02.022

49. Brodke DS, Goz V, Voss MW, Lawrence BD, Spiker WR, Hung M. PROMIS PF CAT Outperforms the ODI and SF-36 Physical Function Domain in Spine Patients. *Spine (Phila Pa 1976)*. 2017;42(12):921-929. doi:10.1097/BRS.0000000000001965

50. Dean DM, Ho BS, Lin A, et al. Predictors of Patient-Reported Function and Pain Outcomes in Operative Ankle Fractures. *Foot Ankle Int*. 2017;38(5):496-501. doi:10.1177/1071100716688176

51. Dowdle SB, Glass N, Anthony CA, Hettrich CM. Use of PROMIS for Patients Undergoing Primary Total Shoulder Arthroplasty. *Orthop J Sports Med*. 2017;5(9):2325967117726044. Published 2017 Sep 15. doi:10.1177/2325967117726044

52. Hancock KJ, Glass N, Anthony CA, et al. Performance of PROMIS for Healthy Patients Undergoing Meniscal Surgery. *J Bone Joint Surg Am*. 2017;99(11):954-958. doi:10.2106/JBJS.16.00848

53. Henn RF 3rd, Dubina AG, Jauregui JJ, Smuda MP, Tracy JK. The Maryland Orthopaedic Registry (MOR): Design and baseline characteristics of a prospective registry. *J Clin Orthop Trauma*. 2017;8(4):301-307. doi:10.1016/j.jcot.2017.04.003

54. Hung M, Saltzman CL, Greene T, et al. The responsiveness of the PROMIS instruments and the qDASH in an upper extremity population. *J Patient Rep Outcomes*. 2017;1(1):12. doi:10.1186/s41687-017-0019-0

55. Hung M, Voss MW, Bounsanga J, Crum AB, Tyser AR. Examination of the PROMIS upper extremity item bank. *J Hand Ther*. 2017;30(4):485-490. doi:10.1016/j.jht.2016.10.008

56. Kaat AJ, Rothrock NE, Vrahas MS, et al. Longitudinal Validation of the PROMIS Physical Function Item Bank in Upper Extremity Trauma. *J Orthop Trauma*. 2017;31(10):e321-e326. doi:10.1097/BOT.0000000000000924

57. Kazmers NH, Hung M, Rane AA, Bounsanga J, Weng C, Tyser AR. Association of Physical Function, Anxiety, and Pain Interference in Nonshoulder Upper Extremity Patients Using the PROMIS Platform. *J Hand Surg Am*. 2017;42(10):781-787. doi:10.1016/j.jhsa.2017.05.008

58. Kleimeyer JP, Wood KB, Lønne G, et al. Surgery for Refractory Coccygodynia: Operative Versus Nonoperative Treatment. *Spine (Phila Pa 1976)*. 2017;42(16):1214-1219. doi:10.1097/BRS.0000000000002053

59. Muppavarapu RC, Tsytsikova L, Gottschalk MB, Dalwadi PP, Cassidy C. Functional Outcomes of 24-Hour Thumb Immobilization in Healthy Volunteers: Prospective Comparative Study. *J Wrist Surg*. 2017;6(3):201-205. doi:10.1055/s-0037-1598025

60. Nixon DC, McCormick JJ, Johnson JE, Klein SE. PROMIS Pain Interference and Physical Function Scores Correlate With the Foot and Ankle Ability Measure (FAAM) in Patients With Hallux Valgus. *Clin Orthop Relat Res*. 2017;475(11):2775-2780. doi:10.1007/s11999-017-5476-5

61. Nota SP, Russchen MJ, Raskin KA, Mankin HJ, Hornicek FJ, Schwab JH. Functional and oncological outcome after surgical resection of the scapula and clavicle for primary chondrosarcoma. *Musculoskelet Surg*. 2017;101(1):67-73. doi:10.1007/s12306-016-0437-9

62. Paulino Pereira NR, Janssen SJ, Raskin KA, et al. Most efficient questionnaires to measure quality of life, physical function, and pain in patients with metastatic spine disease: a cross-sectional prospective survey study. *Spine J*. 2017;17(7):953-961. doi:10.1016/j.spinee.2017.02.006

63. Purvis TE, Andreou E, Neuman BJ, Riley LH 3rd, Skolasky RL. Concurrent Validity and Responsiveness of PROMIS Health Domains Among Patients Presenting for Anterior Cervical Spine Surgery. *Spine (Phila Pa 1976)*. 2017;42(23):E1357-E1365. doi:10.1097/BRS.0000000000002347

64. Schuring N, Aoki H, Gray J, Kerkhoffs GMMJ, Lambert M, Gouttebarge V. Osteoarthritis is associated with symptoms of common mental disorders among former elite athletes. *Knee Surg Sports Traumatol Arthrosc*. 2017;25(10):3179-3185. doi:10.1007/s00167-016-4255-2

65. Sheean AJ, Schmitz MR, Ward CL, et al. Assessment of Disability Related to Femoroacetabular Impingement Syndrome by Use of the Patient-Reported Outcome Measure Information System (PROMIS) and Objective Measures of Physical Performance. *Am J Sports Med*. 2017;45(11):2476-2482. doi:10.1177/0363546517708793

66. St John MJ, Mitten D, Hammert WC. Efficacy of PROMIS Pain Interference and Likert Pain Scores to Assess Physical Function. *J Hand Surg Am*. 2017;42(9):705-710. doi:10.1016/j.jhsa.2017.06.004

67. van der Vliet QM, Paulino Pereira NR, Janssen SJ, et al. What Factors are Associated With Quality Of Life, Pain Interference, Anxiety, and Depression in Patients With Metastatic Bone Disease?. *Clin Orthop Relat Res*. 2017;475(2):498-507. doi:10.1007/s11999-016-5118-3

68. van Wulfften Palthe OD, Houdek MT, Rose PS, et al. How Does the Level of Nerve Root Resection in En Bloc Sacrectomy Influence Patient-Reported Outcomes?. *Clin Orthop Relat Res*. 2017;475(3):607-616. doi:10.1007/s11999-016-4794-3

69. van Wulfften Palthe ODR, Janssen SJ, Wunder JS, et al. What questionnaires to use when measuring quality of life in sacral tumor patients: the updated sacral tumor survey. *Spine J*. 2017;17(5):636-644. doi:10.1016/j.spinee.2016.11.004

70. Anderson MR, Baumhauer JF, DiGiovanni BF, et al. Determining Success or Failure After Foot and Ankle Surgery Using Patient Acceptable Symptom State (PASS) and Patient Reported Outcome Information System (PROMIS). *Foot Ankle Int*. 2018;39(8):894-902. doi:10.1177/1071100718769666

71. Austin DC, Torchia MT, Moschetti WE, Jevsevar DS, Keeney BJ. Patient Outcomes After Total Knee Arthroplasty in Patients Older Than 80 Years. *J Arthroplasty*. 2018;33(11):3465-3473. doi:10.1016/j.arth.2018.07.012

72. Babington JR, Edwards A, Wright AK, Dykstra T, Friedman AS, Sethi RK. Patient-Reported Outcome Measures: Utility for Predicting Spinal Surgery in an Integrated Spine Practice. *PM R*. 2018;10(7):724-729. doi:10.1016/j.pmrj.2017.12.004

73. Bao MH, Keeney BJ, Moschetti WE, Paddock NG, Jevsevar DS. Resident Participation is Not Associated With Worse Outcomes After TKA. *Clin Orthop Relat Res*. 2018;476(7):1375-1390. doi:10.1007/s11999.0000000000000002

74. Beleckas CM, Wright M, Prather H, Chamberlain A, Guattery J, Calfee RP. Relative Prevalence of Anxiety and Depression in Patients With Upper Extremity Conditions. *J Hand Surg Am*. 2018;43(6):571.e1-571.e8. doi:10.1016/j.jhsa.2017.12.006

75. Beleckas CM, Guattery J, Chamberlain AM, Khan T, Kelly MP, Calfee RP. Using Patient-reported Outcomes Measurement Information System Measures to Understand the Relationship Between Improvement in Physical Function and Depressive Symptoms. *J Am Acad Orthop Surg*. 2018;26(24):e511-e518. doi:10.5435/JAAOS-D-17-00039

76. Beleckas CM, Prather H, Guattery J, Wright M, Kelly M, Calfee RP. Anxiety in the orthopedic patient: using PROMIS to assess mental health. *Qual Life Res*. 2018;27(9):2275-2282. doi:10.1007/s11136-018-1867-7

77. Bernholt D, Wright RW, Matava MJ, Brophy RH, Bogunovic L, Smith MV. Patient Reported Outcomes Measurement Information System Scores Are Responsive to Early Changes in Patient Outcomes Following Arthroscopic Partial Meniscectomy. *Arthroscopy*. 2018;34(4):1113-1117. doi:10.1016/j.arthro.2017.10.047

78. Blank AT, Lerman DM, Shaw S, et al. PROMIS^®^ scores in operative metastatic bone disease patients: A multicenter, prospective study. *J Surg Oncol*. 2018;118(3):532-535. doi:10.1002/jso.25159

79. Cavallero M, Rosales R, Caballero J, Virkus WW, Kempton LB, Gaski GE. Locking Plate Fixation in a Series of Bicondylar Tibial Plateau Fractures Raises Treatment Costs Without Clinical Benefit. *J Orthop Trauma*. 2018;32(7):333-337. doi:10.1097/BOT.0000000000001188

80. Chen RE, Papuga MO, Voloshin I, et al. Preoperative PROMIS Scores Predict Postoperative Outcomes After Primary ACL Reconstruction. *Orthop J Sports Med*. 2018;6(5):2325967118771286. Published 2018 May 8. doi:10.1177/2325967118771286

81. Fischerauer SF, Talaei-Khoei M, Vissers FL, Chen N, Vranceanu AM. Pain anxiety differentially mediates the association of pain intensity with function depending on level of intolerance of uncertainty. *J Psychiatr Res*. 2018;97:30-37. doi:10.1016/j.jpsychires.2017.11.006

82. Foucher KC, Cinnamon CC, Ryan CA, Chmell SJ, Dapiton K. Hip abductor strength and fatigue are associated with activity levels more than 1 year after total hip replacement. *J Orthop Res*. 2018;36(5):1519-1525. doi:10.1002/jor.23783

83. Fram B, Wall LB, Gelberman RH, Goldfarb CA. Surgical transposition for chronic instability of the extensor carpi ulnaris tendon. *J Hand Surg Eur Vol*. 2018;43(9):925-930. doi:10.1177/1753193418773036

84. Gausden EB, Levack AE, Sin DN, et al. Validating the Patient Reported Outcomes Measurement Information System (PROMIS) computerized adaptive tests for upper extremity fracture care. *J Shoulder Elbow Surg*. 2018;27(7):1191-1197. doi:10.1016/j.jse.2018.01.014

85. Givens DL, Eskildsen S, Taylor KE, Faldowski RA, Del Gaizo DJ. Timed Up and Go test is predictive of Patient-Reported Outcomes Measurement Information System physical function in patients awaiting total knee arthroplasty. *Arthroplast Today*. 2018;4(4):505-509. Published 2018 Sep 1. doi:10.1016/j.artd.2018.07.010

86. Goode AP, Taylor SS, Hastings SN, Stanwyck C, Coffman CJ, Allen KD. Effects of a Home-Based Telephone-Supported Physical Activity Program for Older Adult Veterans With Chronic Low Back Pain. *Phys Ther*. 2018;98(5):369-380. doi:10.1093/ptj/pzy026

87. Guattery JM, Dardas AZ, Kelly M, Chamberlain A, McAndrew C, Calfee RP. Floor Effect of PROMIS Depression CAT Associated With Hasty Completion in Orthopaedic Surgery Patients. *Clin Orthop Relat Res*. 2018;476(4):696-703. doi:10.1007/s11999.0000000000000076

88. Haskell A, Kim T. Implementation of Patient-Reported Outcomes Measurement Information System Data Collection in a Private Orthopedic Surgery Practice. *Foot Ankle Int*. 2018;39(5):517-521. doi:10.1177/1071100717753967

89. Howard L, Berdusco R, Momoli F, et al. Open reduction internal fixation vs non-operative management in proximal humerus fractures: a prospective, randomized controlled trial protocol. *BMC Musculoskelet Disord*. 2018;19(1):299. Published 2018 Aug 18. doi:10.1186/s12891-018-2223-3

90. Hung M, Voss MW, Bounsanga J, Gu Y, Granger EK, Tashjian RZ. Psychometrics of the Patient-Reported Outcomes Measurement Information System Physical Function instrument administered by computerized adaptive testing and the Disabilities of Arm, Shoulder and Hand in the orthopedic elbow patient population. *J Shoulder Elbow Surg*. 2018;27(3):515-522. doi:10.1016/j.jse.2017.10.015

91. Hung M, Bounsanga J, Voss MW, Saltzman CL. Establishing minimum clinically important difference values for the Patient-Reported Outcomes Measurement Information System Physical Function, hip disability and osteoarthritis outcome score for joint reconstruction, and knee injury and osteoarthritis outcome score for joint reconstruction in orthopaedics. *World J Orthop*. 2018;9(3):41-49. Published 2018 Mar 18. doi:10.5312/wjo.v9.i3.41

92. Hung M, Saltzman CL, Greene T, et al. Evaluating instrument responsiveness in joint function: The HOOS JR, the KOOS JR, and the PROMIS PF CAT. *J Orthop Res*. 2018;36(4):1178-1184. doi:10.1002/jor.23739

93. Hung M, Saltzman CL, Kendall R, et al. What Are the MCIDs for PROMIS, NDI, and ODI Instruments Among Patients With Spinal Conditions?. *Clin Orthop Relat Res*. 2018;476(10):2027-2036. doi:10.1097/CORR.0000000000000419

94. Jildeh TR, Lizzio VA, Meta F, Fidai MS, Kaat AJ, Makhni EC. The Correlation Between PROMIS Pain Interference and VAS Pain in Ambulatory Orthopedic Patients. *Orthopedics*. 2018;41(6):e813-e819. doi:10.3928/01477447-20180912-06

95. Jones IA, Wilson M, Togashi R, Han B, Mircheff AK, Thomas Vangsness C Jr. A randomized, controlled study to evaluate the efficacy of intra-articular, autologous adipose tissue injections for the treatment of mild-to-moderate knee osteoarthritis compared to hyaluronic acid: a study protocol. *BMC Musculoskelet Disord*. 2018;19(1):383. Published 2018 Oct 24. doi:10.1186/s12891-018-2300-7

96. Kadri O, Jildeh TR, Meldau JE, et al. How Long Does It Take for Patients to Complete PROMIS Scores?: An Assessment of PROMIS CAT Questionnaires Administered at an Ambulatory Sports Medicine Clinic. *Orthop J Sports Med*. 2018;6(8):2325967118791180. Published 2018 Aug 14. doi:10.1177/2325967118791180

97. Kagan R, Anderson MB, Christensen JC, Peters CL, Gililland JM, Pelt CE. The Recovery Curve for the Patient-Reported Outcomes Measurement Information System Patient-Reported Physical Function and Pain Interference Computerized Adaptive Tests After Primary Total Knee Arthroplasty. *J Arthroplasty*. 2018;33(8):2471-2474. doi:10.1016/j.arth.2018.03.020

98. Karns MR, Jones DL, Todd DC, et al. Patient- and Procedure-Specific Variables Driving Total Direct Costs of Outpatient Anterior Cruciate Ligament Reconstruction. *Orthop J Sports Med*. 2018;6(8):2325967118788543. Published 2018 Aug 6. doi:10.1177/2325967118788543

99. Kendall R, Wagner B, Brodke D, et al. The Relationship of PROMIS Pain Interference and Physical Function Scales. *Pain Med*. 2018;19(9):1720-1724. doi:10.1093/pm/pnx310

100. Kohring JM, Erickson JA, Anderson MB, Gililland JM, Peters CL, Pelt CE. Treated Versus Untreated Depression in Total Joint Arthroplasty Impacts Outcomes. *J Arthroplasty*. 2018;33(7S):S81-S85. doi:10.1016/j.arth.2018.01.065

101. Kohring JM, Pelt CE, Anderson MB, Peters CL, Gililland JM. Press Ganey Outpatient Medical Practice Survey Scores Do Not Correlate With Patient-Reported Outcomes After Primary Joint Arthroplasty. *J Arthroplasty*. 2018;33(8):2417-2422. doi:10.1016/j.arth.2018.03.044

102. Kootstra TJM, Wilkens SC, Menendez ME, Ring D. Is Physician Empathy Associated With Differences in Pain and Functional Limitations After a Hand Surgeon Visit?. *Clin Orthop Relat Res*. 2018;476(4):801-807. doi:10.1007/s11999.0000000000000077

103. Mastboom MJ, Planje R, van de Sande MA. The Patient Perspective on the Impact of Tenosynovial Giant Cell Tumors on Daily Living: Crowdsourcing Study on Physical Function and Quality of Life. *Interact J Med Res*. 2018;7(1):e4. Published 2018 Feb 23. doi:10.2196/ijmr.9325

104. Merrill RK, Zebala LP, Peters C, Qureshi SA, McAnany SJ. Impact of Depression on Patient-Reported Outcome Measures After Lumbar Spine Decompression. *Spine (Phila Pa 1976)*. 2018;43(6):434-439. doi:10.1097/BRS.0000000000002329

105. Minoughan CE, Schumaier AP, Fritch JL, Grawe BM. Correlation of Patient-Reported Outcome Measurement Information System Physical Function Upper Extremity Computer Adaptive Testing, With the American Shoulder and Elbow Surgeons Shoulder Assessment Form and Simple Shoulder Test in Patients With Shoulder Pain. *Arthroscopy*. 2018;34(5):1430-1436. doi:10.1016/j.arthro.2017.11.040

106. Minoughan CE, Schumaier AP, Fritch JL, Grawe BM. Correlation of PROMIS Physical Function Upper Extremity Computer Adaptive Test with American Shoulder and Elbow Surgeons shoulder assessment form and Simple Shoulder Test in patients with shoulder arthritis. *J Shoulder Elbow Surg*. 2018;27(4):585-591. doi:10.1016/j.jse.2017.10.036

107. Nixon DC, Cusworth BM, McCormick JJ, Johnson JE, Klein SE. Patient-Reported Allergies Do Not Predict Poorer PROMIS Function, Pain, and Depression Scores Following Foot and Ankle Surgery. *Foot Ankle Int*. 2018;39(8):949-953. doi:10.1177/1071100718769667

108. Owen RJ, Zebala LP, Peters C, McAnany S. PROMIS Physical Function Correlation With NDI and mJOA in the Surgical Cervical Myelopathy Patient Population. *Spine (Phila Pa 1976)*. 2018;43(8):550-555. doi:10.1097/BRS.0000000000002373

109. Patel AA, Dodwad SM, Boody BS, et al. Validation of Patient Reported Outcomes Measurement Information System (PROMIS) Computer Adaptive Tests (CATs) in the Surgical Treatment of Lumbar Spinal Stenosis. *Spine (Phila Pa 1976)*. 2018;43(21):1521-1528. doi:10.1097/BRS.0000000000002648

110. Patterson BM, Orvets ND, Aleem AW, et al. Correlation of Patient-Reported Outcomes Measurement Information System (PROMIS) scores with legacy patient-reported outcome scores in patients undergoing rotator cuff repair. *J Shoulder Elbow Surg*. 2018;27(6S):S17-S23. doi:10.1016/j.jse.2018.03.023

111. Purvis TE, Neuman BJ, Riley LH 3rd, Skolasky RL. Discriminant Ability, Concurrent Validity, and Responsiveness of PROMIS Health Domains Among Patients With Lumbar Degenerative Disease Undergoing Decompression With or Without Arthrodesis. *Spine (Phila Pa 1976)*. 2018;43(21):1512-1520. doi:10.1097/BRS.0000000000002661

112. Saad MA, Kassam HF, Suriani RJ Jr, Pan SD, Blaine TA, Kovacevic D. Performance of PROMIS Global-10 compared with legacy instruments in patients with shoulder arthritis. *J Shoulder Elbow Surg*. 2018;27(12):2249-2256. doi:10.1016/j.jse.2018.06.006

113. Scott EJ, Westermann R, Glass NA, Hettrich C, Wolf BR, Bollier MJ. Performance of the PROMIS in Patients After Anterior Cruciate Ligament Reconstruction. *Orthop J Sports Med*. 2018;6(5):2325967118774509. Published 2018 May 25. doi:10.1177/2325967118774509

114. Talaei-Khoei M, Chen N, Ring D, Vranceanu AM. Satisfaction with life moderates the indirect effect of pain intensity on pain interference through pain catastrophizing. *J Consult Clin Psychol*. 2018;86(3):231-241. doi:10.1037/ccp0000283

115. Talaei-Khoei M, Fischerauer SF, Jha R, Ring D, Chen N, Vranceanu AM. Bidirectional mediation of depression and pain intensity on their associations with upper extremity physical function. *J Behav Med*. 2018;41(3):309-317. doi:10.1007/s10865-017-9891-6

116. Tyser AR, Gaffney CJ, Zhang C, Presson AP. The Association of Patient Satisfaction with Pain, Anxiety, and Self-Reported Physical Function. *J Bone Joint Surg Am*. 2018;100(21):1811-1818. doi:10.2106/JBJS.17.00372

117. van der Vliet QMJ, Hietbrink F, Casari F, Leenen LPH, Heng M. Factors Influencing Functional Outcomes of Subtalar Fusion for Posttraumatic Arthritis After Calcaneal Fracture. *Foot Ankle Int*. 2018;39(9):1062-1069. doi:10.1177/1071100718777492

118. Virkus WW, Caballero J, Kempton LB, Cavallero M, Rosales R, Gaski GE. Costs and Complications of Single-Stage Fixation Versus 2-Stage Treatment of Select Bicondylar Tibial Plateau Fractures. *J Orthop Trauma*. 2018;32(7):327-332. doi:10.1097/BOT.0000000000001167

119. Wilkens SC, Lans J, Bargon CA, Ring D, Chen NC. Hand Posturing Is a Nonverbal Indicator of Catastrophic Thinking for Finger, Hand, or Wrist Injury. *Clin Orthop Relat Res*. 2018;476(4):706-713. doi:10.1007/s11999.0000000000000089

120. Wojahn RD, Bogunovic L, Brophy RH, et al. Opioid Consumption After Knee Arthroscopy. *J Bone Joint Surg Am*. 2018;100(19):1629-1636. doi:10.2106/JBJS.18.00049

121. Zdziarski-Horodyski L, Horodyski M, Sadasivan KK, et al. An integrated-delivery-of-care approach to improve patient reported physical function and mental wellbeing after orthopedic trauma: study protocol for a randomized controlled trial. *Trials*. 2018;19(1):32. Published 2018 Jan 11. doi:10.1186/s13063-017-2430-5

122. Alexander JH, Jordan SW, West JM, et al. Targeted muscle reinnervation in oncologic amputees: Early experience of a novel institutional protocol. *J Surg Oncol*. 2019;120(3):348-358. doi:10.1002/jso.25586

123. Alvarez-Nebreda ML, Heng M, Rosner B, et al. Reliability of Proxy-reported Patient-reported Outcomes Measurement Information System Physical Function and Pain Interference Responses for Elderly Patients With Musculoskeletal Injury. *J Am Acad Orthop Surg*. 2019;27(4):e156-e165. doi:10.5435/JAAOS-D-17-00644

124. Austin DC, Torchia MT, Moschetti WE, Jevsevar DS, Keeney BJ. Patient outcomes after total hip arthroplasty in extreme elderly patients older than 80 years. *Hip Int*. 2020;30(4):407-416. doi:10.1177/1120700019837943

125. Austin DC, Torchia MT, Werth PM, Lucas AP, Moschetti WE, Jevsevar DS. A One-Question Patient-Reported Outcome Measure Is Comparable to Multiple-Question Measures in Total Knee Arthroplasty Patients. *J Arthroplasty*. 2019;34(12):2937-2943. doi:10.1016/j.arth.2019.07.023

126. Bakhsh W, Childs S, Oh I, Flemister S, Baumhauer J, Ketz J. Evaluating Patients for Elective Outpatient Foot and Ankle Surgery: Insurance as a Predictor of Patient Outcomes. *Foot Ankle Spec*. 2019;12(6):522-529. doi:10.1177/1938640018823070

127. Beleckas CM, Gerull W, Wright M, Guattery J, Calfee RP. Variability of PROMIS Scores Across Hand Conditions. *J Hand Surg Am*. 2019;44(3):186-191.e1. doi:10.1016/j.jhsa.2018.10.029

128. Beletsky A, Nwachukwu BU, Manderle BJ, et al. The Impact of Workers' Compensation on Patient-Reported Outcomes Measurement Information System Upper Extremity and Legacy Outcome Measures in Patients Undergoing Arthroscopic Rotator Cuff Repair. *Arthroscopy*. 2019;35(10):2817-2824. doi:10.1016/j.arthro.2019.05.027

129. Bernstein DN, Fear K, Mesfin A, et al. Patient-reported outcomes use during orthopaedic surgery clinic visits improves the patient experience. *Musculoskeletal Care*. 2019;17(1):120-125. doi:10.1002/msc.1379

130. Bernstein DN, Houck JR, Mahmood B, Hammert WC. Minimal Clinically Important Differences for PROMIS Physical Function, Upper Extremity, and Pain Interference in Carpal Tunnel Release Using Region- and Condition-Specific PROM Tools. *J Hand Surg Am*. 2019;44(8):635-640. doi:10.1016/j.jhsa.2019.04.004

131. Bernstein DN, Crijns TJ, Mahmood B, Ring D, Hammert WC. Patient Characteristics, Treatment, and Presenting PROMIS Scores Associated with Number of Office Visits for Traumatic Hand and Wrist Conditions. *Clin Orthop Relat Res*. 2019;477(10):2345-2355. doi:10.1097/CORR.0000000000000742

132. Bernstein DN, Mayo K, Baumhauer JF, Dasilva C, Fear K, Houck JR. Do Patient Sociodemographic Factors Impact the PROMIS Scores Meeting the Patient-Acceptable Symptom State at the Initial Point of Care in Orthopaedic Foot and Ankle Patients?. *Clin Orthop Relat Res*. 2019;477(11):2555-2565. doi:10.1097/CORR.0000000000000866

133. Bernstein DN, Anderson MR, Baumhauer JF, et al. A Comparative Analysis of Clinical Outcomes in Noninsertional Versus Insertional Tendinopathy Using PROMIS [published correction appears in Foot Ankle Spec. 2021 Apr 13;:19386400211009676]. *Foot Ankle Spec*. 2019;12(4):350-356. doi:10.1177/1938640018806662

134. Bernstein DN, Kelly M, Houck JR, et al. PROMIS Pain Interference Is Superior vs Numeric Pain Rating Scale for Pain Assessment in Foot and Ankle Patients. *Foot Ankle Int*. 2019;40(2):139-144. doi:10.1177/1071100718803314

135. Bernstein DN, Houck JR, Mahmood B, Hammert WC. Responsiveness of the PROMIS and its Concurrent Validity with Other Region- and Condition-specific PROMs in Patients Undergoing Carpal Tunnel Release. *Clin Orthop Relat Res*. 2019;477(11):2544-2551. doi:10.1097/CORR.0000000000000773

136. Bernstein DN, Houck JR, Hammert WC. A Comparison of PROMIS UE Versus PF: Correlation to PROMIS PI and Depression, Ceiling and Floor Effects, and Time to Completion. *J Hand Surg Am*. 2019;44(10):901.e1-901.e7. doi:10.1016/j.jhsa.2018.12.006

137. Bernstein DN, Bakhsh W, Papuga MO, Menga EN, Rubery PT, Mesfin A. An Evaluation of PROMIS in Patients With Primary or Metastatic Spine Tumors. *Spine (Phila Pa 1976)*. 2019;44(10):747-752. doi:10.1097/BRS.0000000000002934

138. Bernstein DN, Papuga MO, Sanders JO, Rubery PT, Menga EN, Mesfin A. Evaluating the Correlation and Performance of PROMIS to SRS Questionnaires in Adult and Pediatric Spinal Deformity Patients. *Spine Deform*. 2019;7(1):118-124. doi:10.1016/j.jspd.2018.05.010

139. Bernstein DN, St John M, Rubery PT, Mesfin A. PROMIS Pain Interference Is Superior to the Likert Pain Scale for Pain Assessment in Spine Patients. *Spine (Phila Pa 1976)*. 2019;44(14):E852-E856. doi:10.1097/BRS.0000000000002979

140. Bernstein DN, Atkinson J, Fear K, et al. Determining the Generalizability of the PROMIS Depression Domain's Floor Effect and Completion Time in Patients Undergoing Orthopaedic Surgery. *Clin Orthop Relat Res*. 2019;477(10):2215-2225. doi:10.1097/CORR.0000000000000782

141. Bhashyam AR, Jupiter JB. Revision Fixation of Distal Humerus Fracture Nonunions in Older Age Patients with Poor Bone Quality or Bone Loss - Is This Viable as a Long-term Treatment Option?. *Arch Bone Jt Surg*. 2019;7(3):251-257.

142. Bhatt S, Boody BS, Savage JW, Hsu WK, Rothrock NE, Patel AA. Validation of Patient-reported Outcomes Measurement Information System Computer Adaptive Tests in Lumbar Disk Herniation Surgery. *J Am Acad Orthop Surg*. 2019;27(3):95-103. doi:10.5435/JAAOS-D-17-00300

143. Blackburn CW, Thompson NR, Tanenbaum JE, Passerallo AJ, Mroz TE, Steinmetz MP. Association of Cost Savings and Surgical Quality With Single-Vendor Procurement for Spinal Implants. *JAMA Netw Open*. 2019;2(11):e1915567. Published 2019 Nov 1. doi:10.1001/jamanetworkopen.2019.15567

144. Blanchett JW, Kuhlmann NA, Fidai MS, Borowsky PA, Muh SJ, Makhni EC. Using Patient-Reported Outcome Measurement Information System Computer Adaptive Testing Domains to Investigate the Impact of Obesity on Physical Function, Pain Interference, and Mental Health in Sports Medicine Patients. *J Obes Metab Syndr*. 2019;28(4):246-253. doi:10.7570/jomes.2019.28.4.246

145. Brodell JD Jr, MacDonald A, Perkins JA, Deland JT, Oh I. Deltoid-Spring Ligament Reconstruction in Adult Acquired Flatfoot Deformity With Medial Peritalar Instability. *Foot Ankle Int*. 2019;40(7):753-761. doi:10.1177/1071100719839176

146. Carender CN, Bollier MJ, Wolf BR, Duchman KR, An Q, Westermann RW. Preoperative Performance of PROMIS in Patients With Patellofemoral Malalignment and Chondral Disease. *Orthop J Sports Med*. 2019;7(7):2325967119855001. Published 2019 Jul 10. doi:10.1177/2325967119855001

147. Chen RE, Papuga MO, Nicandri GT, Miller RJ, Voloshin I. Preoperative Patient-Reported Outcomes Measurement Information System (PROMIS) scores predict postoperative outcome in total shoulder arthroplasty patients. *J Shoulder Elbow Surg*. 2019;28(3):547-554. doi:10.1016/j.jse.2018.08.040

148. Crijns TJ, Bernstein DN, Ring D, Gonzalez R, Wilbur D, Hammert WC. Factors Associated With a Discretionary Upper-Extremity Surgery. *J Hand Surg Am*. 2019;44(2):155.e1-155.e7. doi:10.1016/j.jhsa.2018.04.028

149. Dumanian GA, Potter BK, Mioton LM, et al. Targeted Muscle Reinnervation Treats Neuroma and Phantom Pain in Major Limb Amputees: A Randomized Clinical Trial. *Ann Surg*. 2019;270(2):238-246. doi:10.1097/SLA.0000000000003088

150. Fidai MS, Tramer JS, Meldau J, et al. Mental Health and Tobacco Use Are Correlated With Physical Function Outcomes in Patients With Knee Pain and Injury. *Arthroscopy*. 2019;35(12):3295-3301. doi:10.1016/j.arthro.2019.06.038

151. Fisk F, Franovic S, Tramer JS, et al. PROMIS CAT forms demonstrate responsiveness in patients following arthroscopic rotator cuff repair across numerous health domains. *J Shoulder Elbow Surg*. 2019;28(12):2427-2432. doi:10.1016/j.jse.2019.04.055

152. Gholson JJ, Shah AS, Buckwalter JA 4th, Buckwalter JA 5th. Long-Term Clinical and Radiographic Follow-Up of Preaxial Polydactyly Reconstruction. *J Hand Surg Am*. 2019;44(3):244.e1-244.e6. doi:10.1016/j.jhsa.2018.05.030

153. Gulbrandsen TR, Khazi ZM, Bollier M, et al. Preoperative Performance of Patient-Reported Outcomes Measurement Information System in Patients with Meniscal Root Tears. *J Knee Surg*. 2021;34(9):913-917. doi:10.1055/s-0039-3402076

154. Gulledge CM, Smith DG, Ziedas A, Muh SJ, Moutzouros V, Makhni EC. Floor and Ceiling Effects, Time to Completion, and Question Burden of PROMIS CAT Domains Among Shoulder and Knee Patients Undergoing Nonoperative and Operative Treatment. *JB JS Open Access*. 2019;4(4):e0015.1-7. Published 2019 Dec 5. doi:10.2106/JBJS.OA.19.00015

155. Hadlandsmyth K, Dindo LN, St Marie BJ, et al. Patient-Reported Outcomes Measurement Information System (PROMIS) Instruments: Reliability and Validity in Veterans Following Orthopedic Surgery. *Eval Health Prof*. 2020;43(4):207-212. doi:10.1177/0163278719856406

156. Hancock KJ, Glass N, Anthony CA, et al. PROMIS: a valid and efficient outcomes instrument for patients with ACL tears. *Knee Surg Sports Traumatol Arthrosc*. 2019;27(1):100-104. doi:10.1007/s00167-018-5034-z

157. Houck J, Kang D, Cuddeford T, Rahkola S. Ability of Patient-Reported Outcomes to Characterize Patient Acceptable Symptom State (PASS) After Attending a Primary Care Physical Therapist and Medical Doctor Collaborative Service: A Cross-Sectional Study. *Arch Phys Med Rehabil*. 2019;100(1):60-66. doi:10.1016/j.apmr.2018.07.443

158. Huang CH, Foucher KC. Step Length Asymmetry and Its Associations With Mechanical Energy Exchange, Function, and Fatigue After Total Hip Replacement. *J Orthop Res*. 2019;37(7):1563-1570. doi:10.1002/jor.24296

159. Hung M, Baumhauer JF, Licari FW, Bounsanga J, Voss MW, Saltzman CL. Responsiveness of the PROMIS and FAAM Instruments in Foot and Ankle Orthopedic Population. *Foot Ankle Int*. 2019;40(1):56-64. doi:10.1177/1071100718799758

160. Hung M, Baumhauer JF, Licari FW, Voss MW, Bounsanga J, Saltzman CL. PROMIS and FAAM Minimal Clinically Important Differences in Foot and Ankle Orthopedics. *Foot Ankle Int*. 2019;40(1):65-73. doi:10.1177/1071100718800304

161. Hung M, Saltzman CL, Voss MW, et al. Responsiveness of the Patient-Reported Outcomes Measurement Information System (PROMIS), Neck Disability Index (NDI) and Oswestry Disability Index (ODI) instruments in patients with spinal disorders. *Spine J*. 2019;19(1):34-40. doi:10.1016/j.spinee.2018.06.355

162. Iyer S, Koltsov JCB, Steinhaus M, et al. A Prospective, Psychometric Validation of National Institutes of Health Patient-Reported Outcomes Measurement Information System Physical Function, Pain Interference, and Upper Extremity Computer Adaptive Testing in Cervical Spine Patients: Successes and Key Limitations. *Spine (Phila Pa 1976)*. 2019;44(22):1539-1549. doi:10.1097/BRS.0000000000003133

163. Janssen SJ, Pereira NRP, Thio QCBS, et al. Physical function and pain intensity in patients with metastatic bone disease. *J Surg Oncol*. 2019;120(3):376-381. doi:10.1002/jso.25510

164. Jayakumar P, Teunis T, Williams M, Lamb SE, Ring D, Gwilym S. Factors associated with the magnitude of limitations during recovery from a fracture of the proximal humerus: predictors of limitations after proximal humerus fracture. *Bone Joint J*. 2019;101-B(6):715-723. doi:10.1302/0301-620X.101B6.BJJ-2018-0857.R1

165. Jayakumar P, Teunis T, Vranceanu AM, Lamb S, Ring D, Gwilym S. Relationship Between Magnitude of Limitations and Patient Experience During Recovery from Upper-Extremity Fracture. *JB JS Open Access*. 2019;4(3):e0002.1-7. Published 2019 Jul 22. doi:10.2106/JBJS.OA.19.00002

166. Jayakumar P, Teunis T, Vranceanu AM, et al. Construct Validity and Precision of Different Patient-reported Outcome Measures During Recovery After Upper Extremity Fractures. *Clin Orthop Relat Res*. 2019;477(11):2521-2530. doi:10.1097/CORR.0000000000000928

167. Jevotovsky DS, Thirukumaran CP, Rubery PT. Creating value in spine surgery: using patient reported outcomes to compare the short-term impact of different orthopedic surgical procedures. *Spine J*. 2019;19(11):1850-1857. doi:10.1016/j.spinee.2019.05.595

168. Johnson B, Stekas N, Ayres E, et al. PROMIS Correlates With Legacy Outcome Measures in Patients With Neck Pain and Improves Upon NDI When Assessing Disability in Cervical Deformity. *Spine (Phila Pa 1976)*. 2019;44(14):982-988. doi:10.1097/BRS.0000000000002994

169. Kaat AJ, Buckenmaier CT 3rd, Cook KF, et al. The expansion and validation of a new upper extremity item bank for the Patient-Reported Outcomes Measurement Information System® (PROMIS). *J Patient Rep Outcomes*. 2019;3(1):69. Published 2019 Nov 26. doi:10.1186/s41687-019-0158-6

170. Kahan JB, Kassam HF, Nicholson AD, Saad MA, Kovacevic D. Performance of PROMIS Global-10 to Legacy Instruments in Patients With Lateral Epicondylitis. *Arthroscopy*. 2019;35(3):770-774. doi:10.1016/j.arthro.2018.09.019

171. Katarzyna K, Glinkowski WM. Patient-reported outcomes of carpal tunnel syndrome surgery in a non-industrial area. *Ann Agric Environ Med*. 2019;26(2):350-354. doi:10.26444/aaem/99004

172. Kazmers NH, Stephens AR, Tyser AR. Effects of Baseline Opioid Medication Use on Patient-Reported Functional and Psychological Impairment Among Hand Clinic Patients. *J Hand Surg Am*. 2019;44(10):829-839. doi:10.1016/j.jhsa.2019.07.003

173. Kazmers NH, Hung M, Bounsanga J, Voss MW, Howenstein A, Tyser AR. Minimal Clinically Important Difference After Carpal Tunnel Release Using the PROMIS Platform. *J Hand Surg Am*. 2019;44(11):947-953.e1. doi:10.1016/j.jhsa.2019.03.006

174. Keeney BJ, Austin DC, Jevsevar DS. Preoperative Weight Loss for Morbidly Obese Patients Undergoing Total Knee Arthroplasty: Determining the Necessary Amount. *J Bone Joint Surg Am*. 2019;101(16):1440-1450. doi:10.2106/JBJS.18.01136

175. Kelly MP, Kallen MA, Shaffrey CI, et al. Examining the Patient-Reported Outcomes Measurement Information System versus the Scoliosis Research Society-22r in adult spinal deformity [published online ahead of print, 2019 Feb 22]. *J Neurosurg Spine*. 2019;1-6. doi:10.3171/2018.11.SPINE181014

176. Kenney RJ, Houck J, Giordano BD, Baumhauer JF, Herbert M, Maloney MD. Do Patient Reported Outcome Measurement Information System (PROMIS) Scales Demonstrate Responsiveness as Well as Disease-Specific Scales in Patients Undergoing Knee Arthroscopy?. *Am J Sports Med*. 2019;47(6):1396-1403. doi:10.1177/0363546519832546

177. Khechen B, Patel DV, Haws BE, et al. Evaluating the Concurrent Validity of PROMIS Physical Function in Anterior Cervical Discectomy and Fusion. *Clin Spine Surg*. 2019;32(10):449-453. doi:10.1097/BSD.0000000000000786

178. Khechen B, Haws BE, Patel DV, et al. PROMIS Physical Function Score Strongly Correlates With Legacy Outcome Measures in Minimally Invasive Lumbar Microdiscectomy. *Spine (Phila Pa 1976)*. 2019;44(6):442-446. doi:10.1097/BRS.0000000000002841

179. Kohan EM, Hill JR, Schwabe M, Aleem AW, Keener JD, Chamberlain AM. The influence of mental health on Patient-Reported Outcomes Measurement Information System (PROMIS) and traditional outcome instruments in patients with symptomatic glenohumeral arthritis. *J Shoulder Elbow Surg*. 2019;28(2):e40-e48. doi:10.1016/j.jse.2018.07.033

180. Kollmorgen RC, Hutyra CA, Green C, Lewis B, Olson SA, Mather RC 3rd. Relationship Between PROMIS Computer Adaptive Tests and Legacy Hip Measures Among Patients Presenting to a Tertiary Care Hip Preservation Center. *Am J Sports Med*. 2019;47(4):876-884. doi:10.1177/0363546518825252

181. Kortlever JTP, Leyton-Mange A, Keulen MHF, et al. PROMIS Physical Function Correlates with KOOS, JR in Patients with Knee Pain. *J Knee Surg*. 2020;33(9):903-911. doi:10.1055/s-0039-1688780

182. Lans J, Chebib IA, Castelein RM, Chen NC, Lozano-Calderón S. Reconstruction of the Proximal Aspect of the Radius After Desmoplastic Fibroma Resection: A Case Report. *JBJS Case Connect*. 2019;9(1):e12. doi:10.2106/JBJS.CC.18.00158

183. Lansdown DA, Morrison C, Zaid MB, et al. Preoperative IDEAL (Iterative Decomposition of Echoes of Asymmetrical Length) magnetic resonance imaging rotator cuff muscle fat fractions are associated with rotator cuff repair outcomes. *J Shoulder Elbow Surg*. 2019;28(10):1936-1941. doi:10.1016/j.jse.2019.05.018

184. Lazaridou A, Martel MO, Cornelius M, et al. The Association Between Daily Physical Activity and Pain Among Patients with Knee Osteoarthritis: The Moderating Role of Pain Catastrophizing. *Pain Med*. 2019;20(5):916-924. doi:10.1093/pm/pny129

185. Łazarski A, Sarzyńska S, Struzik S, Jędral T, Łęgosz P, Małdyk P. Results of Treatment of Type 3 Acromioclavicular Joint Dislocation with Three Methods. *Ortop Traumatol Rehabil*. 2019;21(3):167-179. doi:10.5604/01.3001.0013.2921

186. Livermore AT, Anderson LA, Anderson MB, Erickson JA, Peters CL. Correction of mildly dysplastic hips with periacetabular osteotomy demonstrates promising outcomes, achievement of correction goals, and excellent five-year survivorship. *Bone Joint J*. 2019;101-B(6_Supple_B):16-22. doi:10.1302/0301-620X.101B6.BJJ-2018-1487.R1

187. Lizzio VA, Blanchett J, Borowsky P, et al. Feasibility of PROMIS CAT Administration in the Ambulatory Sports Medicine Clinic With Respect to Cost and Patient Compliance: A Single-Surgeon Experience. *Orthop J Sports Med*. 2019;7(1):2325967118821875. Published 2019 Jan 22. doi:10.1177/2325967118821875

188. Magaldi RJ, Staff I, Stovall AE, Stohler SA, Lewis CG. Impact of Resilience on Outcomes of Total Knee Arthroplasty. *J Arthroplasty*. 2019;34(11):2620-2623.e1. doi:10.1016/j.arth.2019.06.008

189. Mahmood B, Chongshu C, Qiu X, Messing S, Hammert WC. Comparison of the Michigan Hand Outcomes Questionnaire, Boston Carpal Tunnel Questionnaire, and PROMIS Instruments in Carpal Tunnel Syndrome. *J Hand Surg Am*. 2019;44(5):366-373. doi:10.1016/j.jhsa.2018.10.031

190. Makhni EC, Meldau JE, Blanchett J, et al. Correlation of PROMIS Physical Function, Pain Interference, and Depression in Pediatric and Adolescent Patients in the Ambulatory Sports Medicine Clinic. *Orthop J Sports Med*. 2019;7(6):2325967119851100. Published 2019 Jun 24. doi:10.1177/2325967119851100

191. Manoli A 3rd, Markel JF, Pizzimenti NM, Markel DC. Early Results of a Modern Uncemented Total Knee Arthroplasty System. *Orthopedics*. 2019;42(6):355-360. doi:10.3928/01477447-20190906-04

192. Martusiewicz A, Delagrammaticas D, Harold RE, Bhatt S, Beal MD, Manning DW. Anterior versus posterior approach total hip arthroplasty: patient-reported and functional outcomes in the early postoperative period. *Hip Int*. 2020;30(6):695-702. doi:10.1177/1120700019881413

193. Massier JRA, Ogink PT, Schlösser TPC, et al. Sagittal spinal parameters after en bloc resection of mobile spine tumors. *Spine J*. 2019;19(10):1606-1612. doi:10.1016/j.spinee.2019.05.012

194. Medina SH, Nadarajah V, Jauregui JJ, et al. Orthopaedic surgery patients who use recreational marijuana have less pre-operative pain. *Int Orthop*. 2019;43(2):283-292. doi:10.1007/s00264-018-4101-x

195. Meldau JE, Borowsky P, Blanchett J, et al. Impact of Patient Demographic Factors on Preoperative Patient-Reported Outcomes Measurement Information System (PROMIS) Physical Function, Pain Interference, and Depression Computer Adaptive Testing Scores in Patients Undergoing Shoulder and Elbow Surgery. *Orthop J Sports Med*. 2019;7(11):2325967119884543. Published 2019 Nov 22. doi:10.1177/2325967119884543

196. Miles M, Nadarajah V, Jauregui JJ, et al. Evaluation of the PROMIS Physical Function Computer Adaptive Test in Patients Undergoing Knee Surgery. *J Knee Surg*. 2020;33(8):810-817. doi:10.1055/s-0039-1688691

197. Monroe EJ, Flores SE, Chambers CC, et al. Patient-Reported Outcomes After Isolated and Combined Arthroscopic Subscapularis Tendon Repairs. *Arthroscopy*. 2019;35(6):1779-1784. doi:10.1016/j.arthro.2019.01.034

198. Moses MJ, Tishelman JC, Stekas N, et al. Comparison of Patient Reported Outcome Measurement Information System With Neck Disability Index and Visual Analog Scale in Patients With Neck Pain. *Spine (Phila Pa 1976)*. 2019;44(3):E162-E167. doi:10.1097/BRS.0000000000002796

199. Nadarajah V, Glazier E, Miller K, et al. Evaluation of Preoperative Pain Using PROMIS Pain Interference in Knee Surgery Patients. *J Knee Surg*. 2020;33(9):875-883. doi:10.1055/s-0039-1688769

200. Ngan A, Xiao W, Curran PF, et al. Functional workspace and patient-reported outcomes improve after reverse and total shoulder arthroplasty. *J Shoulder Elbow Surg*. 2019;28(11):2121-2127. doi:10.1016/j.jse.2019.03.029

201. Nicholson AD, Kassam HF, Pan SD, Berman JE, Blaine TA, Kovacevic D. Performance of PROMIS Global-10 Compared With Legacy Instruments for Rotator Cuff Disease. *Am J Sports Med*. 2019;47(1):181-188. doi:10.1177/0363546518810508

202. Nixon DC, Schafer KA, Cusworth B, McCormick JJ, Johnson JE, Klein SE. Preoperative Anxiety Effect on Patient-Reported Outcomes Following Foot and Ankle Surgery. *Foot Ankle Int*. 2019;40(9):1007-1011. doi:10.1177/1071100719850806

203. Nwachukwu BU, Beck EC, Chapman R, Chahla J, Okoroha K, Nho SJ. Preoperative Performance of the PROMIS in Patients Undergoing Hip Arthroscopic Surgery for Femoroacetabular Impingement Syndrome. *Orthop J Sports Med*. 2019;7(7):2325967119860079. Published 2019 Jul 29. doi:10.1177/2325967119860079

204. Owen RJ, Khan AZ, McAnany SJ, Peters C, Zebala LP. PROMIS correlation with NDI and VAS measurements of physical function and pain in surgical patients with cervical disc herniations and radiculopathy [published online ahead of print, 2019 Jul 5]. *J Neurosurg Spine*. 2019;1-6. doi:10.3171/2019.4.SPINE18422

205. Özkan S, Teunis T, Ring DC, Chen NC. What Is the Effect of Vitamin C on Finger Stiffness After Distal Radius Fracture? A Double-blind, Placebo-controlled Randomized Trial. *Clin Orthop Relat Res*. 2019;477(10):2278-2286. doi:10.1097/CORR.0000000000000807

206. Padilla JA, Rudy HL, Gabor JA, et al. Relationship Between the Patient-Reported Outcome Measurement Information System and Traditional Patient-Reported Outcomes for Osteoarthritis [published correction appears in J Arthroplasty. 2019 Mar 6;:]. *J Arthroplasty*. 2019;34(2):265-272. doi:10.1016/j.arth.2018.10.012

207. Palsgrove A, Patton C, King P, Gelfand J, Turcotte J. A comparison of PROMIS Global Health-Mental and legacy orthopedic outcome measures for evaluating preoperative mental health status [published correction appears in J Orthop. 2020 Dec 15;24:293]. *J Orthop*. 2019;19:98-101. Published 2019 Nov 27. doi:10.1016/j.jor.2019.11.032

208. Pennings JS, Devin CJ, Khan I, Bydon M, Asher AL, Archer KR. Prediction of Oswestry Disability Index (ODI) using PROMIS-29 in a national sample of lumbar spine surgery patients. *Qual Life Res*. 2019;28(10):2839-2850. doi:10.1007/s11136-019-02223-8

209. Phillips JLH, Warrender WJ, Lutsky KF, Beredjiklian PK. Evaluation of the PROMIS Upper Extremity Computer Adaptive Test Against Validated Patient-Reported Outcomes in Patients With Basilar Thumb Arthritis. *J Hand Surg Am*. 2019;44(7):564-569. doi:10.1016/j.jhsa.2019.01.003

210. Pierce KE, Alas H, Brown AE, et al. PROMIS physical health domain scores are related to cervical deformity severity. *J Craniovertebr Junction Spine*. 2019;10(3):179-183. doi:10.4103/jcvjs.JCVJS_52_19

211. Ploetze KL, Dalton JF, Calfee RP, McDonald DJ, O'Keefe RJ, Cipriano CA. Patient-Reported Outcomes Measurement Information System physical function correlates with Toronto Extremity Salvage Score in an orthopaedic oncology population. *J Orthop Translat*. 2019;19:143-150. Published 2019 Mar 8. doi:10.1016/j.jot.2019.02.004

212. Raad M, Neuman BJ, Kebaish KM, Riley LH 3rd, Skolasky RL. Estimating Health Utility in Patients Presenting for Spine Surgery Using Patient-reported Outcomes Measurement Information System (PROMIS) Health Domains. *Spine (Phila Pa 1976)*. 2019;44(13):908-914. doi:10.1097/BRS.0000000000002977

213. Raad M, Jain A, Huang M, et al. Validity and responsiveness of PROMIS in adult spinal deformity: The need for a self-image domain. *Spine J*. 2019;19(1):50-55. doi:10.1016/j.spinee.2018.07.014

214. Rojas EO, Glass N, Owens J, et al. Performance of the PROMIS in Patients Undergoing 3 Common Elbow Procedures. *Orthop J Sports Med*. 2019;7(6):2325967119852595. Published 2019 Jun 17. doi:10.1177/2325967119852595

215. Rothrock NE, Bass M, Blumenthal A, et al. AO Patient Outcomes Center: Design, Implementation, and Evaluation of a Software Application for the Collection of Patient-Reported Outcome Measures in Orthopedic Outpatient Clinics. *JMIR Form Res*. 2019;3(2):e10880. Published 2019 Apr 12. doi:10.2196/10880

216. Rothrock NE, Kaat AJ, Vrahas MS, et al. Validation of PROMIS Physical Function Instruments in Patients With an Orthopaedic Trauma to a Lower Extremity. *J Orthop Trauma*. 2019;33(8):377-383. doi:10.1097/BOT.0000000000001493

217. Rubery PT, Houck J, Mesfin A, Molinari R, Papuga MO. Preoperative Patient Reported Outcomes Measurement Information System Scores Assist in Predicting Early Postoperative Success in Lumbar Discectomy. *Spine (Phila Pa 1976)*. 2019;44(5):325-333. doi:10.1097/BRS.0000000000002823

218. Sandvall B, Okoroafor UC, Gerull W, Guattery J, Calfee RP. Minimal Clinically Important Difference for PROMIS Physical Function in Patients With Distal Radius Fractures. *J Hand Surg Am*. 2019;44(6):454-459.e1. doi:10.1016/j.jhsa.2019.02.015

219. Schwartz CE, Zhang J, Rapkin BD, Finkelstein JA. Reconsidering the minimally important difference: evidence of instability over time and across groups. *Spine J*. 2019;19(4):726-734. doi:10.1016/j.spinee.2018.09.010

220. Shah J, Titus AJ, OʼToole RV, et al. Are Geriatric Patients Who Sustain High-Energy Traumatic Injury Likely to Return to Functional Independence?. *J Orthop Trauma*. 2019;33(5):234-238. doi:10.1097/BOT.0000000000001436

221. Shim J, Hamilton DF. Comparative responsiveness of the PROMIS-10 Global Health and EQ-5D questionnaires in patients undergoing total knee arthroplasty. *Bone Joint J*. 2019;101-B(7):832-837. doi:10.1302/0301-620X.101B7.BJJ-2018-1543.R1

222. Squires MD, Brodke DS, Neese A, et al. Physical function computer adaptive test outcomes in diabetic lumbar spine surgical patients. *Spine J*. 2019;19(6):1048-1056. doi:10.1016/j.spinee.2018.12.008

223. Steinhaus ME, Iyer S, Lovecchio F, et al. Minimal Clinically Important Difference and Substantial Clinical Benefit Using PROMIS CAT in Cervical Spine Surgery. *Clin Spine Surg*. 2019;32(9):392-397. doi:10.1097/BSD.0000000000000895

224. Steinhaus ME, Iyer S, Lovecchio F, et al. Which NDI domains best predict change in physical function in patients undergoing cervical spine surgery?. *Spine J*. 2019;19(10):1698-1705. doi:10.1016/j.spinee.2019.06.006

225. Stephan A, Mainzer J, Kümmel D, Impellizzeri FM. Measurement properties of PROMIS short forms for pain and function in orthopedic foot and ankle surgery patients. *Qual Life Res*. 2019;28(10):2821-2829. doi:10.1007/s11136-019-02221-w

226. Stevens KN, Nadarajah V, Jauregui JJ, et al. Preoperative Expectations of Patients Undergoing Knee Surgery. *J Knee Surg*. 2021;34(6):612-620. doi:10.1055/s-0039-1698805

227. Stiegel KR, Lash JG, Peace AJ, Coleman MM, Harrington MA, Cahill CW. Early Experience and Results Using Patient-Reported Outcomes Measurement Information System Scores in Primary Total Hip and Knee Arthroplasty. *J Arthroplasty*. 2019;34(10):2313-2318. doi:10.1016/j.arth.2019.05.044

228. Strong B, Maloney M, Baumhauer J, et al. Psychometric evaluation of the Patient-Reported Outcomes Measurement Information System (PROMIS) Physical Function and Pain Interference Computer Adaptive Test for subacromial impingement syndrome. *J Shoulder Elbow Surg*. 2019;28(2):324-329. doi:10.1016/j.jse.2018.07.024

229. Tyser AR, Hung M, Bounsanga J, Voss MW, Kazmers NH. Evaluation of Version 2.0 of the PROMIS Upper Extremity Computer Adaptive Test in Nonshoulder Upper Extremity Patients. *J Hand Surg Am*. 2019;44(4):267-273. doi:10.1016/j.jhsa.2019.01.008

230. Valerio IL, Dumanian GA, Jordan SW, et al. Preemptive Treatment of Phantom and Residual Limb Pain with Targeted Muscle Reinnervation at the Time of Major Limb Amputation. *J Am Coll Surg*. 2019;228(3):217-226. doi:10.1016/j.jamcollsurg.2018.12.015

231. Verhiel SHWL, Greenberg J, Zale EL, Chen NC, Ring DC, Vranceanu AM. What Role Does Positive Psychology Play in Understanding Pain Intensity and Disability Among Patients with Hand and Upper Extremity Conditions?. *Clin Orthop Relat Res*. 2019;477(8):1769-1776. doi:10.1097/CORR.0000000000000694

232. Wilke B, Cooper A, Scarborough M, Gibbs P, Spiguel A. A Comparison of Limb Salvage Versus Amputation for Nonmetastatic Sarcomas Using Patient-reported Outcomes Measurement Information System Outcomes. *J Am Acad Orthop Surg*. 2019;27(8):e381-e389. doi:10.5435/JAAOS-D-17-00758

233. Wilke BK, Cooper AR, Aratani AK, Scarborough MT, Gibbs CP, Spiguel A. Evaluation of Planned versus Unplanned Soft-Tissue Sarcoma Resection Using PROMIS Measures. *Sarcoma*. 2019;2019:1342615. Published 2019 Mar 5. doi:10.1155/2019/1342615

234. Wilke B, Cooper A, Scarborough M, Gibbs CP, Spiguel A. An Evaluation of PROMIS Health Domains in Sarcoma Patients Compared to the United States Population. *Sarcoma*. 2019;2019:9725976. Published 2019 Jan 16. doi:10.1155/2019/9725976

235. Wright MA, Beleckas CM, Calfee RP. Mental and Physical Health Disparities in Patients With Carpal Tunnel Syndrome Living With High Levels of Social Deprivation. *J Hand Surg Am*. 2019;44(4):335.e1-335.e9. doi:10.1016/j.jhsa.2018.05.019

236. Wright MA, Adelani M, Dy C, OʼKeefe R, Calfee RP. What is the Impact of Social Deprivation on Physical and Mental Health in Orthopaedic Patients?. *Clin Orthop Relat Res*. 2019;477(8):1825-1835. doi:10.1097/CORR.0000000000000698

237. Yoo JS, Parrish JM, Jenkins NW, et al. PROMIS PF in the Evaluation of Postoperative Outcomes in Workers' Compensation Patients Following Anterior Cervical Discectomy and Fusion. *Clin Spine Surg*. 2020;33(7):E312-E316. doi:10.1097/BSD.0000000000000927

238. Yoo JS, Hrynewycz NM, Brundage TS, Singh K. The Use of Patient-Reported Outcome Measurement Information System Physical Function to Predict Outcomes Based on Body Mass Index Following Minimally Invasive Transforaminal Lumbar Interbody Fusion. *Spine (Phila Pa 1976)*. 2019;44(23):E1388-E1395. doi:10.1097/BRS.0000000000003137

239. Abdurrob A, Smith JT. The Effect of Health Insurance Coverage on Orthopaedic Patient-reported Outcome Measures. *J Am Acad Orthop Surg*. 2020;28(16):e729-e734. doi:10.5435/JAAOS-D-19-00487

240. Anantavorasakul N, Lans J, Macken AA, Sood RF, Chen NC, Eberlin KR. Surgery for lower extremity symptomatic neuroma: Long-term outcomes. *J Plast Reconstr Aesthet Surg*. 2020;73(8):1456-1464. doi:10.1016/j.bjps.2020.01.034

241. Aneizi A, Sajak PMJ, Alqazzaz A, et al. Impact of Preoperative Opioid Use on 2-Year Patient-Reported Outcomes in Knee Surgery Patients [published online ahead of print, 2020 Sep 8]. *J Knee Surg*. 2020;10.1055/s-0040-1716358. doi:10.1055/s-0040-1716358

242. Beletsky A, Lu Y, Nwachukwu BU, et al. Preoperative psychometric properties of visual analog scale asessments for function, pain, and strength compared with legacy upper extremity outcome measures in glenohumeral osteoarthritis. *JSES Int*. 2020;4(3):443-448. Published 2020 Jun 17. doi:10.1016/j.jseint.2020.03.006

243. Beletsky A, Nwachukwu BU, Gorodischer T, et al. Psychometric properties of visual analog scale assessments for function, pain, and strength compared with disease-specific upper extremity outcome measures in rotator cuff repair. *JSES Int*. 2020;4(3):619-624. Published 2020 May 23. doi:10.1016/j.jseint.2020.04.012

244. Bernstein DN, Greenstein AS, D'Amore T, Mesfin A. Do PROMIS Physical Function, Pain Interference, and Depression Correlate to the Oswestry Disability Index and Neck Disability Index in Spine Trauma Patients?. *Spine (Phila Pa 1976)*. 2020;45(11):764-769. doi:10.1097/BRS.0000000000003376

245. Bhashyam AR, Ochen Y, van der Vliet QMJ, et al. Association of Patient-reported Outcomes With Clinical Outcomes After Distal Humerus Fracture Treatment. *J Am Acad Orthop Surg Glob Res Rev*. 2020;4(2):e19.00122. Published 2020 Feb 6. doi:10.5435/JAAOSGlobal-D-19-00122

246. Brodell JD Jr, Ayers BC, Baumhauer JF, et al. Chopart Amputation: Questioning the Clinical Efficacy of a Long-standing Surgical Option for Diabetic Foot Infection. *J Am Acad Orthop Surg*. 2020;28(16):684-691. doi:10.5435/JAAOS-D-19-00757

247. Carney J, Ton A, Alluri RK, Grisdela P, Marecek GS. Complications following operative treatment of supination-adduction type II (AO/OTA 44A2.3) ankle fractures. *Injury*. 2020;51(6):1387-1391. doi:10.1016/j.injury.2020.03.032

248. Chavez JL, Porucznik CA, Gren LH, et al. The Impact of Preoperative Mindfulness-Based Stress Reduction on Postoperative Outcomes in Lumbar Spine Degenerative Disease: 3-Month and 12-Month Results of a Pilot Study. *World Neurosurg*. 2020;139:e230-e236. doi:10.1016/j.wneu.2020.03.186

249. Chen RE, Brown AM, Greenstein AS, Miller RJ, Mannava S, Voloshin I. Cemented versus uncemented fixation of second-generation Trabecular Metal glenoid components: minimum 5-year outcomes. *J Shoulder Elbow Surg*. 2021;30(4):e147-e156. doi:10.1016/j.jse.2020.07.025

250. Cheng AL, Fogarty AE, Calfee RP, Salter A, Colditz GA, Prather H. Differences in Self-Reported Physical and Behavioral Health in Musculoskeletal Patients Based on Physician Gender. *PM R*. 2021;13(7):720-728. doi:10.1002/pmrj.12468

251. Chrea B, Eble SK, Day J, Ellis SJ, Drakos MC; HSS Orthopaedic Foot and Ankle Surgery Group. Comparison Between Polyvinyl Alcohol Implant and Cheilectomy With Moberg Osteotomy for Hallux Rigidus. *Foot Ankle Int*. 2020;41(9):1031-1040. doi:10.1177/1071100720947380

252. Coxe FR, Wessel LE, Verret CI, Stepan JG, Nguyen JT, Fufa DT. Impact of Patient-Reported Allergies on Early Postoperative Opioid Use and Outcomes Following Ambulatory Hand Surgery [published online ahead of print, 2020 Jun 7]. *Hand (N Y)*. 2020;1558944720928483. doi:10.1177/1558944720928483

253. Crijns TJ, Bernstein DN, Gonzalez R, Wilbur D, Ring D, Hammert WC. Operative Treatment is Not Associated with More Relief of Depression Symptoms than Nonoperative Treatment in Patients with Common Hand Illness. *Clin Orthop Relat Res*. 2020;478(6):1319-1329. doi:10.1097/CORR.0000000000001170

254. Crijns TJ, Bernstein DN, Teunis T, et al. The Association Between Symptoms of Depression and Office Visits in Patients With Nontraumatic Upper-Extremity Illness. *J Hand Surg Am*. 2020;45(2):159.e1-159.e8. doi:10.1016/j.jhsa.2019.03.019

255. Davies JP, Ma X, Garfinkel J, et al. Subtalar Fusion for Correction of Forefoot Abduction in Stage II Adult-Acquired Flatfoot Deformity [published online ahead of print, 2020 Aug 24]. *Foot Ankle Spec*. 2020;1938640020951050. doi:10.1177/1938640020951050

256. Day J, de Cesar Netto C, Nishikawa DRC, et al. Three-Dimensional Biometric Weightbearing CT Evaluation of the Operative Treatment of Adult-Acquired Flatfoot Deformity. *Foot Ankle Int*. 2020;41(8):930-936. doi:10.1177/1071100720925423

257. DiLiberto FE, Aslan DH, Houck JR, Ho BS, Vora AM, Haddad SL. Overall Health and the Influence of Physical Therapy on Physical Function Following Total Ankle Arthroplasty. *Foot Ankle Int*. 2020;41(11):1383-1390. doi:10.1177/1071100720942473

258. Drayer NJ, Wallace CS, Yu HH, et al. High Resiliency Linked to Short-Term Patient Reported Outcomes and Return to Duty Following Arthroscopic Knee Surgery. *Mil Med*. 2020;185(1-2):112-116. doi:10.1093/milmed/usz180

259. Eble SK, Hansen OB, Chrea B, et al. Clinical Outcomes of the Polyvinyl Alcohol (PVA) Hydrogel Implant for Hallux Rigidus. *Foot Ankle Int*. 2020;41(9):1056-1064. doi:10.1177/1071100720932526

260. Kollmorgen RC, Hutyra CA, Green C, Lewis B, Olson SA, Mather RC 3rd. Relationship Between PROMIS Computer Adaptive Tests and Legacy Hip Measures Among Patients Presenting to a Tertiary Care Hip Preservation Center. *Am J Sports Med*. 2019;47(4):876-884. doi:10.1177/0363546518825252

261. Gilley J, Bell R, Lima M, et al. Prospective Patient Reported Outcomes (PRO) Study Assessing Outcomes of Surgically Managed Ankle Fractures. *Foot Ankle Int*. 2020;41(2):206-210. doi:10.1177/1071100719891157

262. Giordano NA, Kent M, Buckenmaier CC 3rd, et al. A Longitudinal Comparison of Patient-Reported Outcomes Measurement Information System to Legacy Scales in Knee and Shoulder Arthroscopy Patients. *Arthroscopy*. 2021;37(1):185-194.e2. doi:10.1016/j.arthro.2020.07.026

263. Gire JD, Koltsov JCB, Segovia NA, Kenney DE, Yao J, Ladd AL. Single Assessment Numeric Evaluation (SANE) in Hand Surgery: Does a One-Question Outcome Instrument Compare Favorably?. *J Hand Surg Am*. 2020;45(7):589-596. doi:10.1016/j.jhsa.2020.03.024

264. Grandizio LC, Follett L, Skudalski L, Dwyer CL, Klena JC. The Handshake Test: A Nonverbal Assessment of Coping Strategies and Functional Status in Patients with Atraumatic Upper-Extremity Conditions. *Iowa Orthop J*. 2020;40(1):49-52.

265. Haan EA, Terwee CB, Van Wier MF, et al. Translation, cross-cultural and construct validity of the Dutch-Flemish PROMIS® upper extremity item bank v2.0. *Qual Life Res*. 2020;29(4):1123-1135. doi:10.1007/s11136-019-02388-2

266. Hajewski CJ, Baron JE, Glass NA, et al. Performance of the Patient-Reported Outcome Measurement Information System in Patients With Patellofemoral Instability. *Orthop J Sports Med*. 2020;8(4):2325967120915540. Published 2020 Apr 21. doi:10.1177/2325967120915540

267. Hao SP, Houck JR, Waldman OV, Baumhauer JF, Oh I. Prediction of post-interventional physical function in diabetic foot ulcer patients using patient reported outcome measurement information system (PROMIS). *Foot Ankle Surg*. 2021;27(2):224-230. doi:10.1016/j.fas.2020.04.009

268. Henry LE, Aneizi A, Nadarajah V, et al. Preoperative expectations and early postoperative met expectations of extremity orthopaedic surgery. *J Clin Orthop Trauma*. 2020;11(Suppl 5):S829-S836. doi:10.1016/j.jcot.2020.06.027

269. Howard B, Aneizi A, Nadarajah V, et al. Early patient satisfaction following orthopaedic surgery. *J Clin Orthop Trauma*. 2020;11(Suppl 5):S823-S828. doi:10.1016/j.jcot.2020.06.037

270. Hunt D, Zhang T, Koenig S, et al. Predictors of PROMIS Physical Function at 2 Years following Knee Surgery [published online ahead of print, 2020 Sep 8]. *J Knee Surg*. 2020;10.1055/s-0040-1716360. doi:10.1055/s-0040-1716360

271. Ibaseta A, Rahman R, Skolasky RL, Reidler JS, Kebaish KM, Neuman BJ. SRS-22r legacy scores can be accurately translated to PROMIS scores in adult spinal deformity patients. *Spine J*. 2020;20(2):234-240. doi:10.1016/j.spinee.2019.09.006

272. Jayakumar P, Teunis T, Vranceanu AM, Lamb S, Ring D, Gwilym S. Early Psychological and Social Factors Explain the Recovery Trajectory After Distal Radial Fracture. *J Bone Joint Surg Am*. 2020;102(9):788-795. doi:10.2106/JBJS.19.00100

273. Jayakumar P, Teunis T, Vranceanu AM, et al. The impact of a patient's engagement in their health on the magnitude of limitations and experience following upper limb fractures. *Bone Joint J*. 2020;102-B(1):42-47. doi:10.1302/0301-620X.102B1.BJJ-2019-0421.R1

274. Jenkins NW, Parrish JM, Brundage TS, Hrynewycz NM, Singh K. Association of Preoperative PROMIS Scores With Short-term Postoperative Improvements in Physical Function After Minimally Invasive Transforaminal Lumbar Interbody Fusion. *Neurospine*. 2020;17(2):417-425. doi:10.14245/ns.2040048.024

275. Jenkins NW, Parrish JM, Cha EDK, et al. Validation of PROMIS Physical Function in MIS TLIF: 2-Year Follow-up. *Spine (Phila Pa 1976)*. 2020;45(22):E1516-E1522. doi:10.1097/BRS.0000000000003635

276. Kaiser PB, Newman ET, Haggerty C, et al. A Limited Fixation, Olecranon Sparing Approach, for Management of Geriatric Intra-Articular Distal Humerus Fractures. *Geriatr Orthop Surg Rehabil*. 2020;11:2151459320950063. Published 2020 Aug 14. doi:10.1177/2151459320950063

277. Kazmers NH, Grasu B, Presson AP, Ou Z, Henrie NB, Tyser AR. The Prognostic Value of Preoperative Patient-Reported Function and Psychological Characteristics on Early Outcomes Following Trapeziectomy With Ligament Reconstruction Tendon Interposition for Treatment of Thumb Carpometacarpal Osteoarthritis. *J Hand Surg Am*. 2020;45(6):469-478. doi:10.1016/j.jhsa.2019.11.016

278. Kazmers NH, Qiu Y, Yoo M, Stephens AR, Tyser AR, Zhang Y. The Minimal Clinically Important Difference of the PROMIS and QuickDASH Instruments in a Nonshoulder Hand and Upper Extremity Patient Population. *J Hand Surg Am*. 2020;45(5):399-407.e6. doi:10.1016/j.jhsa.2019.12.002

279. Kempton LB, Schneble CA, Brown K, Sorkin AT, Virkus WW. Significant Improvement in the Value of Surgical Treatment of Tibial Plateau Fractures Through Surgeon Practice Standardization. *J Am Acad Orthop Surg*. 2020;28(18):772-779. doi:10.5435/JAAOS-D-18-00720

280. Khalil LS, Darrith B, Franovic S, Davis JJ, Weir RM, Banka TR. Patient-Reported Outcomes Measurement Information System (PROMIS) Global Health Short Forms Demonstrate Responsiveness in Patients Undergoing Knee Arthroplasty. *J Arthroplasty*. 2020;35(6):1540-1544. doi:10.1016/j.arth.2020.01.032

281. Kim T, Haskell A. Patient-Reported Outcomes After Structural Autograft for Large or Cystic Talar Dome Osteochondral Lesions. *Foot Ankle Int*. 2020;41(5):549-555. doi:10.1177/1071100720907313

282. Klemt C, Tirumala V, Oganesyan R, Xiong L, van den Kieboom J, Kwon YM. Single-Stage Revision of the Infected Total Knee Arthroplasty Is Associated With Improved Functional Outcomes: A Propensity Score-Matched Cohort Study. *J Arthroplasty*. 2021;36(1):298-304. doi:10.1016/j.arth.2020.07.012

283. Kohring JM, Greenstein A, Gorczyca JT, Judd KT, Soles G, Ketz JP. Immediate Improvement in Physical Function After Symptomatic Syndesmotic Screw Removal. *J Orthop Trauma*. 2020;34(6):327-331. doi:10.1097/BOT.0000000000001766

284. Kohring JM, Houck JR, Oh I, Flemister AS, Ketz JP, Baumhauer JF. Pattern of recovery and outcomes of patient reported physical function and pain interference after ankle fusion: a retrospective cohort study. *J Patient Rep Outcomes*. 2020;4(1):40. Published 2020 May 27. doi:10.1186/s41687-020-00203-y

285. Kolade O, Ghosh N, Luthringer TA, et al. Correlation of Patient Reported Outcome Measurement Information System (PROMIS) with American Shoulder and Elbow Surgeon (ASES), and Constant (CS) scores in idiopathic adhesive capsulitis. *J Shoulder Elbow Surg*. 2021;30(3):554-560. doi:10.1016/j.jse.2020.05.040

286. Kortlever JTP, Karyampudi P, Ottenhoff JSE, Ring D, Vagner GA, Reichel LM. Using the Tampa Scale for Kinesiophobia Short Form in Patients With Upper Extremity Specific Limitations [published online ahead of print, 2020 Jan 22]. *Hand (N Y)*. 2020;1558944719898830. doi:10.1177/1558944719898830

287. La A, Nadarajah V, Jauregui JJ, et al. Clinical characteristics associated with depression or anxiety among patients presenting for knee surgery. *J Clin Orthop Trauma*. 2020;11(Suppl 1):S164-S170. doi:10.1016/j.jcot.2019.08.009

288. Lameijer CM, van Bruggen SGJ, Haan EJA, et al. Graded response model fit, measurement invariance and (comparative) precision of the Dutch-Flemish PROMIS® Upper Extremity V2.0 item bank in patients with upper extremity disorders. *BMC Musculoskelet Disord*. 2020;21(1):170. Published 2020 Mar 16. doi:10.1186/s12891-020-3178-8

289. Lanman TH, CuÉllar JM. Restoration of Spinal Motion: Conversion of Anterior Cervical Fusion With Pseudarthrosis to Artificial Disc Replacement. *Int J Spine Surg*. 2020;14(4):483-487. doi:10.14444/7063

290. Lans J, Baker DJ, Castelein RM, Sood RF, Chen NC, Eberlin KR. Patient-Reported Outcomes following Surgical Treatment of Symptomatic Digital Neuromas. *Plast Reconstr Surg*. 2020;145(3):563e-573e. doi:10.1097/PRS.0000000000006552

291. Lawrie CM, Abu-Amer W, Barrack RL, Clohisy JC. Is the Patient-Reported Outcome Measurement Information System Feasible in Bundled Payment for Care Improvement in Total Hip Arthroplasty Patients?. *J Arthroplasty*. 2020;35(5):1179-1185. doi:10.1016/j.arth.2019.12.021

292. Lawrie CM, Abu-Amer WY, Clohisy JC. Is the Patient-Reported Outcome Measurement Information System Feasible in Bundled Payment for Care Improvement Total Knee Arthroplasty Patients?. *J Arthroplasty*. 2021;36(1):6-12. doi:10.1016/j.arth.2020.07.041

293. Lee JH, Cook JL, Wilson N, Rucinski K, Stannard JP. Outcomes after Multiligament Knee Injury Reconstruction using Novel Graft Constructs and Techniques [published online ahead of print, 2020 Sep 25]. *J Knee Surg*. 2020;10.1055/s-0040-1716356. doi:10.1055/s-0040-1716356

294. Li DJ, Clohisy JC, Schwabe MT, Yanik EL, Pascual-Garrido C. PROMIS Versus Legacy Patient-Reported Outcome Measures in Patients Undergoing Surgical Treatment for Symptomatic Acetabular Dysplasia. *Am J Sports Med*. 2020;48(2):385-394. doi:10.1177/0363546519894323

295. Livermore AT, Erickson JA, Blackburn B, Peters CL. Does the sequential addition of accelerometer-based navigation and sensor-guided ligament balancing improve outcomes in TKA?. *Bone Joint J*. 2020;102-B(6_Supple_A):24-30. doi:10.1302/0301-620X.102B6.BJJ-2019-1634.R1

296. Lu Y, Agarwalla A, Patel BH, et al. Relationship between the Patient-Reported Outcomes Measurement Information System (PROMIS) computer adaptive testing and legacy instruments in patients undergoing isolated biceps tenodesis. *J Shoulder Elbow Surg*. 2020;29(6):1214-1222. doi:10.1016/j.jse.2019.11.003

297. MacDonald A, Houck J, Baumhauer JF. Role of Patient-Reported Outcome Measures on Predicting Outcome of Bunion Surgery. *Foot Ankle Int*. 2020;41(2):133-139. doi:10.1177/1071100719886286

298. Metcalf KB, Du JY, Lapite IO, et al. Comparison of Infrapatellar and Suprapatellar Approaches for Intramedullary Nail Fixation of Tibia Fractures. *J Orthop Trauma*. 2021;35(2):e45-e50. doi:10.1097/BOT.0000000000001897

299. Mioton LM, Dumanian GA, Shah N, et al. Targeted Muscle Reinnervation Improves Residual Limb Pain, Phantom Limb Pain, and Limb Function: A Prospective Study of 33 Major Limb Amputees. *Clin Orthop Relat Res*. 2020;478(9):2161-2167. doi:10.1097/CORR.0000000000001323

300. Molloy IB, Yong TM, Keswani A, et al. Do Medicare's Patient-Reported Outcome Measures Collection Windows Accurately Reflect Academic Clinical Practice?. *J Arthroplasty*. 2020;35(4):911-917. doi:10.1016/j.arth.2019.11.006

301. Monroe EJ, Flores SE, Zhang AL, Feeley BT, Lansdown DA, Ma CB. Do Outcomes of Arthroscopic Subscapularis Tendon Repairs Depend on Rotator Cuff Fatty Infiltration?. *Orthop J Sports Med*. 2020;8(4):2325967120913036. Published 2020 Apr 3. doi:10.1177/2325967120913036

302. Nadarajah V, Stevens KN, Henry L, et al. Patients undergoing shoulder surgery have high preoperative expectations. *Knee Surg Sports Traumatol Arthrosc*. 2020;28(7):2377-2385. doi:10.1007/s00167-019-05824-5

303. Nadarajah V, Sood A, Kator JL, et al. Evaluation of preoperative pain in patients undergoing shoulder surgery using the PROMIS pain interference computer-adaptive test. *J Clin Orthop Trauma*. 2020;11(Suppl 4):S539-S545. doi:10.1016/j.jcot.2020.04.025

304. Nayar SK, Glasser R, Deune EG, Ingari JV, LaPorte DM. Equivalent PROMIS Scores after Nonoperative or Operative Treatment of Trapeziometacarpal Osteoarthritis. *Arch Bone Jt Surg*. 2020;8(3):383-390. doi:10.22038/abjs.2019.41772.2128

305. Newman ET, Lans J, Kim J, et al. PROMIS Function Scores Are Lower in Patients Who Underwent More Aggressive Local Treatment for Desmoid Tumors [published correction appears in Clin Orthop Relat Res. 2020 May;478(5):1132. Calderon, Santiago Lozano [corrected to Lozano-Calderon, Santiago A]]. *Clin Orthop Relat Res*. 2020;478(3):563-577. doi:10.1097/CORR.0000000000000918

306. Nixon DC, Zhang C, Weinberg MW, Presson AP, Nickisch F. Relationship of Press Ganey Satisfaction and PROMIS Function and Pain in Foot and Ankle Patients. *Foot Ankle Int*. 2020;41(10):1206-1211. doi:10.1177/1071100720937013

307. Nwachukwu BU, Beletsky A, Naveen N, et al. Patient-Reported Outcomes Measurement Information System (PROMIS) Instruments Correlate Better With Legacy Measures in Knee Cartilage Patients at Postoperative Than at Preoperative Assessment. *Arthroscopy*. 2020;36(5):1419-1428. doi:10.1016/j.arthro.2020.01.036

308. Nwachukwu BU, Rasio J, Beck EC, et al. Patient-Reported Outcomes Measurement Information System Physical Function Has a Lower Effect Size and is Less Responsive Than Legacy Hip Specific Patient Reported Outcome Measures Following Arthroscopic Hip Surgery. *Arthroscopy*. 2020;36(12):2992-2997. doi:10.1016/j.arthro.2020.07.008

309. Ochen Y, Peek J, McTague MF, et al. Long-term outcomes after open reduction and internal fixation of bicondylar tibial plateau fractures. *Injury*. 2020;51(4):1097-1102. doi:10.1016/j.injury.2020.03.003

310. Okoroha KR, Ussef N, Jildeh TR, et al. Comparison of Tendon Lengthening With Traditional Versus Accelerated Rehabilitation After Achilles Tendon Repair: A Prospective Randomized Controlled Trial. *Am J Sports Med*. 2020;48(7):1720-1726. doi:10.1177/0363546520909389

311. Okoroha KR, Lu Y, Nwachukwu BU, et al. How Should We Define Clinically Significant Improvement on Patient-Reported Outcomes Measurement Information System Test for Patients Undergoing Knee Meniscal Surgery?. *Arthroscopy*. 2020;36(1):241-250. doi:10.1016/j.arthro.2019.07.036

312. O'Neill DC, Mortensen AJ, Cannamela PC, Aoki SK. Clinical and Radiographic Presentation of Capsular Iatrogenic Hip Instability After Previous Hip Arthroscopy. *Am J Sports Med*. 2020;48(12):2927-2932. doi:10.1177/0363546520949821

313. Paget LDA, Aoki H, Kemp S, et al. Ankle osteoarthritis and its association with severe ankle injuries, ankle surgeries and health-related quality of life in recently retired professional male football and rugby players: a cross-sectional observational study. *BMJ Open*. 2020;10(6):e036775. Published 2020 Jun 21. doi:10.1136/bmjopen-2020-036775

314. Parrish JM, Jenkins NW, Patel DV, et al. Demographic and Perioperative Factors Associated With Patient-reported Outcomes Measurement Information System (PROMIS) Survey Completion. *Clin Spine Surg*. 2020;33(10):E519-E524. doi:10.1097/BSD.0000000000000998

315. Parrish JM, Jenkins NW, Hrynewycz NM, Brundage TS, Singh K. The Relationship Between Preoperative PROMIS Scores With Postoperative Improvements in Physical Function After Anterior Cervical Discectomy and Fusion. *Neurospine*. 2020;17(2):398-406. doi:10.14245/ns.1938352.176

316. Parrish JM, Jenkins NW, Brundage TS, Hrynewycz NM, Yoo JS, Singh K. PROMIS Physical Function Predicts Postoperative Pain and Disability Following Anterior Cervical Discectomy and Fusion. *Clin Spine Surg*. 2020;33(9):382-387. doi:10.1097/BSD.0000000000000973

317. Parrish JM, Jenkins NW, Hrynewycz NM, Brundage TS, Yoo JS, Singh K. The Impact of Comorbidity Burden on Postoperative PROMIS Physical Function Following Minimally Invasive Transforaminal Lumbar Interbody Fusion. *Clin Spine Surg*. 2020;33(6):E294-E298. doi:10.1097/BSD.0000000000000934

318. Parrish JM, Jenkins NW, Hrynewycz NM, Brundage TS, Singh K. The influence of gender on postoperative PROMIS physical function outcomes following minimally invasive transforaminal lumbar interbody fusion. *J Clin Orthop Trauma*. 2020;11(5):910-915. doi:10.1016/j.jcot.2020.04.007

319. Parrish JM, Jenkins NW, Narain AS, Hrynewycz NM, Brundage TS, Singh K. Postoperative Pain, Narcotics Consumption, and Patient-Reported Outcomes Based on PROMIS Physical Function Following a Single-Level Anterior Cervical Discectomy and Fusion. *Spine (Phila Pa 1976)*. 2020;45(17):E1091-E1096. doi:10.1097/BRS.0000000000003482

320. Passias PG, Horn SR, Segreto FA, et al. ODI Cannot Account for All Variation in PROMIS Scores in Patients With Thoracolumbar Disorders. *Global Spine J*. 2020;10(4):399-405. doi:10.1177/2192568219851478

321. Pennings JS, Khan I, Davidson CA, et al. Using PROMIS-29 to predict Neck Disability Index (NDI) scores using a national sample of cervical spine surgery patients. *Spine J*. 2020;20(8):1305-1315. doi:10.1016/j.spinee.2020.04.028

322. Perraut G, Aneizi A, Nadarajah V, et al. PROMIS physical function two weeks following orthopaedic surgery. *J Clin Orthop Trauma*. 2020;11(Suppl 5):S837-S843. doi:10.1016/j.jcot.2020.06.014

323. Pignolo RJ, Cheung K, Kile S, et al. Self-reported baseline phenotypes from the International Fibrodysplasia Ossificans Progressiva (FOP) Association Global Registry. *Bone*. 2020;134:115274. doi:10.1016/j.bone.2020.115274

324. Plessow F, Pascual-Leone A, McCracken CM, et al. Self-Reported Cognitive Function and Mental Health Diagnoses among Former Professional American-Style Football Players. *J Neurotrauma*. 2020;37(8):1021-1028. doi:10.1089/neu.2019.6661

325. Rijk L, Kortlever JTP, Tipton GW Jr, Ring D, Queralt MV; Austin Spine Study Group. Is It Time to Replace the Oswestry Index With PROMIS Physical Function Computer Adaptive Test?. *Arch Phys Med Rehabil*. 2020;101(9):1549-1555. doi:10.1016/j.apmr.2020.03.021

326. Roberts AM, Voloshin I. Mapping physical functions of the shoulder to American Shoulder and Elbow Surgeons and Patient-Reported Outcomes Measurement Information System scores. *J Shoulder Elbow Surg*. 2020;29(4):707-718. doi:10.1016/j.jse.2019.08.017

327. Sajak PM, Aneizi A, Gopinath R, et al. Factors associated with early postoperative survey completion in orthopaedic surgery patients. *J Clin Orthop Trauma*. 2020;11(Suppl 1):S158-S163. doi:10.1016/j.jcot.2019.07.007

328. Schoenfeld AJ, Blucher JA, Barton LB, et al. Design of the prospective observational study of spinal metastasis treatment (POST). *Spine J*. 2020;20(4):572-579. doi:10.1016/j.spinee.2019.10.021

329. Shamrock AG, Wolf BR, Ortiz SF, et al. Preoperative Validation of the Patient-Reported Outcomes Measurement Information System in Patients With Articular Cartilage Defects of the Knee. *Arthroscopy*. 2020;36(2):516-520. doi:10.1016/j.arthro.2019.08.043

330. Sharma J, Maenza C, Myers A, et al. Clinical Outcomes and Shoulder Kinematics for the "Gray Zone" Extra-articular Scapula Fracture in 5 Patients. *Int J Orthop*. 2020;3(1):1017.

331. Smith E, Klemt C, Sabeh K, Tirumala V, Kwon YM. Patient-reported Outcomes Associated With Preoperative Opioid Use in Revision Total Hip Arthroplasty: A Propensity Score-Matched Cohort Study. *J Am Acad Orthop Surg*. 2021;29(7):e330-e336. doi:10.5435/JAAOS-D-20-00823

332. Speck RM, Ye X, Bernthal NM, Gelhorn HL. Psychometric properties of a custom Patient-Reported Outcomes Measurement Information System (PROMIS) physical function short form and worst stiffness numeric rating scale in tenosynovial giant cell tumors. *J Patient Rep Outcomes*. 2020;4(1):61. Published 2020 Jul 16. doi:10.1186/s41687-020-00217-6

333. Stekas ND, Johnson B, Jevotovsky D, et al. PROMIS is superior to established outcome measures in capturing disability resulting from sagittal malalignment in patients with back pain. *Spine Deform*. 2020;8(3):499-505. doi:10.1007/s43390-020-00068-7

334. Suriani RJ, Kassam HF, Passarelli NR, Esparza R, Kovacevic D. Validation of PROMIS Global-10 compared with legacy instruments in patients with shoulder instability. *Shoulder Elbow*. 2020;12(4):243-252. doi:10.1177/1758573219843617

335. Tamminga SJ, van Vree FM, Volker G, et al. Changes in the ability to participate in and satisfaction with social roles and activities in patients in outpatient rehabilitation. *J Patient Rep Outcomes*. 2020;4(1):73. Published 2020 Sep 1. doi:10.1186/s41687-020-00236-3

336. Torchia MT, Austin DC, Werth PM, Lucas AP, Moschetti WE, Jevsevar DS. A SANE Approach to Outcome Collection? Comparing the Performance of Single- Versus Multiple-Question Patient-Reported Outcome Measures After Total Hip Arthroplasty. *J Arthroplasty*. 2020;35(6S):S207-S213. doi:10.1016/j.arth.2020.01.015

337. Tramer JS, Khalil LS, Fidai MS, et al. Mental health and tobacco use are correlated with PROMIS upper extremity and pain interference scores in patients with shoulder pathology [published online ahead of print, 2020 Jul 13]. *Musculoskelet Surg*. 2020;10.1007/s12306-020-00674-8. doi:10.1007/s12306-020-00674-8

338. Vaishnav AS, McAnany SJ, Iyer S, Albert TJ, Gang CH, Qureshi SA. Psychometric Evaluation of Patient-reported Outcomes Measurement Information System Physical Function Computer Adaptive Testing in Minimally Invasive Lumbar Spine Surgery: An Analysis of Responsiveness, Coverage, Discriminant Validity, and Concurrent Validity. *J Am Acad Orthop Surg*. 2020;28(17):717-729. doi:10.5435/JAAOS-D-19-00306

339. Varlotta CG, Manning JH, Ayres EW, et al. Preoperative MRI predictors of health-related quality of life improvement after microscopic lumbar discectomy. *Spine J*. 2020;20(3):391-398. doi:10.1016/j.spinee.2019.09.020

340. Varlotta C, Fernandez L, Manning J, et al. Evaluation of Health-related Quality of Life Improvement in Patients Undergoing Spine Versus Adult Reconstructive Surgery. *Spine (Phila Pa 1976)*. 2020;45(18):E1179-E1184. doi:10.1097/BRS.0000000000003588

341. Verhiel SHWL, Özkan S, Langhammer CG, Chen NC. The Serially-Operated Essex-Lopresti Injury: Long-Term Outcomes in a Retrospective Cohort. *J Hand Microsurg*. 2020;12(1):47-55. doi:10.1055/s-0039-3401380

342. Virk S, Sandhu M, Qureshi S, Albert T, Sandhu H. How does preoperative opioid use impact postoperative health-related quality of life scores for patients undergoing lumbar microdiscectomy?. *Spine J*. 2020;20(8):1196-1202. doi:10.1016/j.spinee.2020.05.094

343. Virk S, Vaishnav AS, Mok JK, et al. How do high preoperative pain scores impact the clinical course and outcomes for patients undergoing lumbar microdiscectomy? [published online ahead of print, 2020 Aug 7]. *J Neurosurg Spine*. 2020;1-7. doi:10.3171/2020.5.SPINE20373

344. Waldman OV, Hao SP, Houck JR, Lee NJ, Baumhauer JF, Oh I. Operative Intervention Does Not Change Pain Perception in Patients With Diabetic Foot Ulcers. *Clin Diabetes*. 2020;38(2):132-140. doi:10.2337/cd19-0031

345. Warren B, Black N, Linscheid L, Panchbhavi V, Janney C. Use of Ultrasonically Interdigitated Anchors in Haglund's Deformity Correction Surgery. *Foot Ankle Spec*. 2021;14(2):148-152. doi:10.1177/1938640020908455

346. Weiner JA, Snavely JE, Johnson DJ, Hsu WK, Patel AA. Impact of Preoperative Opioid Use on Postoperative Patient-reported Outcomes in Lumbar Spine Surgery Patients. *Clin Spine Surg*. 2021;34(3):E154-E159. doi:10.1097/BSD.0000000000001067

347. Wilkin GP, Poitras S, Clohisy J, et al. Periacetabular osteotomy with or without arthroscopic management in patients with hip dysplasia: study protocol for a multicenter randomized controlled trial. *Trials*. 2020;21(1):725. Published 2020 Aug 18. doi:10.1186/s13063-020-04592-9

348. Yoo JS, Hrynewycz NM, Brundage TS, et al. The Influence of Preoperative Mental Health on PROMIS Physical Function Outcomes Following Minimally Invasive Transforaminal Lumbar Interbody Fusion. *Spine (Phila Pa 1976)*. 2020;45(4):E236-E243. doi:10.1097/BRS.0000000000003236

349. Yoo JS, Jenkins NW, Parrish JM, et al. Evaluation of Postoperative Mental Health Outcomes in Patients Based on Patient-Reported Outcome Measurement Information System Physical Function Following Anterior Cervical Discectomy and Fusion. *Neurospine*. 2020;17(1):184-189. doi:10.14245/ns.1938256.128

350. Zdziarski-Horodyski L, Vasilopoulos T, Horodyski M, et al. Can an Integrative Care Approach Improve Physical Function Trajectories after Orthopaedic Trauma? A Randomized Controlled Trial. *Clin Orthop Relat Res*. 2020;478(4):792-804. doi:10.1097/CORR.0000000000001140

351. Zitsch BP, Stannard JP, Worley JR, Cook JL, Leary EV. Patient-Reported Outcomes for Large Bipolar Osteochondral Allograft Transplantation in Combination with Realignment Osteotomies for the Knee. *J Knee Surg*. 2021;34(11):1260-1266. doi:10.1055/s-0040-1710361

352. Metcalf KB, Ochenjele G. Primary Triple Arthrodesis Equivalent for Complete Extruded Missing Talus with Associated Midfoot Instability: A Case Report. *JBJS Case Connect*. 2020;10(2):e0268. doi:10.2106/JBJS.CC.19.00268

353. Mioton LM, Dumanian GA, Fracol ME, et al. Benchmarking Residual Limb Pain and Phantom Limb Pain in Amputees through a Patient-reported Outcomes Survey. *Plast Reconstr Surg Glob Open*. 2020;8(7):e2977. Published 2020 Jul 15. doi:10.1097/GOX.0000000000002977

354. Guo EW, Elhage K, Cross AG, et al. Establishing and comparing reference preoperative Patient-Reported Outcomes Measurement Information System (PROMIS) scores in patients undergoing shoulder surgery. *J Shoulder Elbow Surg*. 2021;30(6):1223-1229. doi:10.1016/j.jse.2020.09.003

355. Tarabochia M, Menendez ME, Ring D. Health Literacy and Decisional Preferences in Hand Surgery. *J Hand Surg Am*. 2021;46(1):70.e1-70.e8. doi:10.1016/j.jhsa.2020.08.008

356. MacMahon A, Cody EA, Caolo K, et al. Association Between Baseline PROMIS Scores, Patient-Provider Communication Factors, and Musculoskeletal Health Literacy on Patient and Surgeon Expectations in Foot and Ankle Surgery. *Foot Ankle Int*. 2021;42(2):192-199. doi:10.1177/1071100720959017

357. Van Wyngaarden JJ, Noehren B, Pennings JS, Jacobs C, Matuszewski PE, Archer KR. Reliability and Validity Evidence of the STarT-Lower Extremity Screening Tool for Patients With Lower Extremity Fracture: A Prospective Study. *Arch Phys Med Rehabil*. 2021;102(2):261-269. doi:10.1016/j.apmr.2020.08.023

358. Karhade AV, Sisodia RC, Bono CM, et al. Surgeon-level variance in achieving clinical improvement after lumbar decompression: the importance of adequate risk adjustment. *Spine J*. 2021;21(3):405-410. doi:10.1016/j.spinee.2020.10.005

359. Kuhns BD, Reuter J, Lawton D, Kenney RJ, Baumhauer JF, Giordano BD. Threshold Values for Success After Hip Arthroscopy Using the Patient-Reported Outcomes Measurement Information System Assessment: Determining the Minimum Clinically Important Difference and Patient Acceptable Symptomatic State. *Am J Sports Med*. 2020;48(13):3280-3287. doi:10.1177/0363546520960461

360. Kempton LB, Gaski GE, Brown K, McKinley TO, Virkus WW. Predictors of Improved Early Clinical Outcomes After Elective Implant Removal. *J Orthop Trauma*. 2021;35(3):e103-e107. doi:10.1097/BOT.0000000000001920

361. Kortlever JTP, Tripathi S, Ring D, McDonald J, Smoot B, Laverty D. Tampa Scale for Kinesiophobia Short Form and Lower Extremity Specific Limitations. *Arch Bone Jt Surg*. 2020;8(5):581-588. doi:10.22038/abjs.2020.40004.2073

362. Haunschild ED, Gilat R, Fu MC, et al. Establishing the Minimal Clinically Important Difference, Patient Acceptable Symptomatic State, and Substantial Clinical Benefit of the PROMIS Upper Extremity Questionnaire After Rotator Cuff Repair. *Am J Sports Med*. 2020;48(14):3439-3446. doi:10.1177/0363546520964957

363. Karhade AV, Fogel HA, Cha TD, et al. Development of prediction models for clinically meaningful improvement in PROMIS scores after lumbar decompression. *Spine J*. 2021;21(3):397-404. doi:10.1016/j.spinee.2020.10.026

364. Lu Y, Beletsky A, Nwachukwu BU, et al. Performance of PROMIS Physical Function, Pain Interference, and Depression Computer Adaptive Tests Instruments in Patients Undergoing Meniscal Surgery. *Arthrosc Sports Med Rehabil*. 2020;2(5):e451-e459. Published 2020 Oct 24. doi:10.1016/j.asmr.2020.04.012

365. Franovic S, Kuhlmann NA, Pietroski A, et al. Preoperative Patient-Centric Predictors of Postoperative Outcomes in Patients Undergoing Arthroscopic Meniscectomy. *Arthroscopy*. 2021;37(3):964-971. doi:10.1016/j.arthro.2020.10.042

366. Kleiss IIM, Kortlever JTP, Ring D, Vagner GA, Reichel LM. A Randomized Controlled Trial of Decision Aids for Upper-Extremity Conditions. *J Hand Surg Am*. 2021;46(4):338.e1-338.e15. doi:10.1016/j.jhsa.2020.09.003

367. Groot OQ, Paulino Pereira NR, Bongers MER, et al. Do Cohabitants Reliably Complete Questionnaires for Patients in a Terminal Cancer Stage when Assessing Quality of Life, Pain, Depression, and Anxiety?. *Clin Orthop Relat Res*. 2021;479(4):792-801. doi:10.1097/CORR.0000000000001525

368. Bernstein DN, Merchan N, Fear K, Rubery PT, Mesfin A. Greater Socioeconomic Disadvantage Is Associated with Worse Symptom Severity at Initial Presentation in Patients Seeking Care for Lumbar Disc Herniation. *Spine (Phila Pa 1976)*. 2021;46(7):464-471. doi:10.1097/BRS.0000000000003811

369. Cremers T, Zoulfi Khatiri M, van Maren K, Ring D, Teunis T, Fatehi A. Moderators and Mediators of Activity Intolerance Related to Pain. *J Bone Joint Surg Am*. 2021;103(3):205-212. doi:10.2106/JBJS.20.00241

370. Seward MW, Antonelli BJ, Giunta N, et al. Weight loss before total joint arthroplasty using a remote dietitian and mobile app: study protocol for a multicenter randomized, controlled trial. *J Orthop Surg Res*. 2020;15(1):531. Published 2020 Nov 13. doi:10.1186/s13018-020-02059-w

371. Melnic CM, Paschalidis A, Katakam A, Bedair HS, Heng M; MGB Arthroplasty Patient-Reported Outcomes Writing Committee. Patient-Reported Mental Health Score Influences Physical Function After Primary Total Knee Arthroplasty. *J Arthroplasty*. 2021;36(4):1277-1283. doi:10.1016/j.arth.2020.10.031

372. Schreiner AJ, Stannard JP, Cook CR, et al. Initial clinical outcomes comparing frozen versus fresh meniscus allograft transplants. *Knee*. 2020;27(6):1811-1820. doi:10.1016/j.knee.2020.09.015

373. Barnes RH, Shapiro JA, Woody N, Chen F, Olcott CW, Del Gaizo DJ. Reducing Opioid Prescriptions Lowers Consumption Without Detriment to Patient-Reported Pain Interference Scores After Total Hip and Knee Arthroplasties. *Arthroplast Today*. 2020;6(4):919-924. Published 2020 Nov 6. doi:10.1016/j.artd.2020.09.017

374. Bido J, Sullivan SW, Carr JB 2nd, Schairer WW, Nwachukwu BU. PROMIS Global-10 performs poorly relative to legacy shoulder instruments in patients undergoing total shoulder arthroplasty for glenohumeral arthritis. *J Shoulder Elbow Surg*. 2021;30(8):1780-1786. doi:10.1016/j.jse.2020.10.021

375. O'Hara NN, Kringos DS, Slobogean GP, Degani Y, Klazinga NS. Patients Place More of an Emphasis on Physical Recovery Than Return to Work or Financial Recovery. *Clin Orthop Relat Res*. 2021;479(6):1333-1343. doi:10.1097/CORR.0000000000001583

376. Wilkinson JT, Clawson JW, Allen CM, Presson AP, Tyser AR, Kazmers NH. Reliability of Telephone Acquisition of the PROMIS Upper Extremity Computer Adaptive Test. *J Hand Surg Am*. 2021;46(3):187-199. doi:10.1016/j.jhsa.2020.09.014

377. Cochrane SK, Calfee RP, Stonner MM, Dale AM. The relationship between depression, anxiety, and pain interference with therapy referral and utilization among patients with hand conditions [published online ahead of print, 2020 Oct 15]. *J Hand Ther*. 2020;S0894-1130(20)30185-X. doi:10.1016/j.jht.2020.10.006

378. Cochrane S, Dale AM, Buckner-Petty S, Sobel AD, Lippold B, Calfee RP. Relevance of Diagnosed Depression and Antidepressants to PROMIS Depression Scores Among Hand Surgical Patients. *J Hand Surg Am*. 2021;46(2):99-105. doi:10.1016/j.jhsa.2020.10.012

379. Oyer MA, Edelstein AI, Arnett NF, Hardt KD, Manning DW, Stover MD. Assessment of Psychological Factors in Short-Stay Total Hip Arthroplasty Protocol. *J Arthroplasty*. 2021;36(4):1336-1341. doi:10.1016/j.arth.2020.10.056

380. Friedman JM, You JS, Hodax JD, et al. Patellar tendon reconstruction with hamstring autograft for the treatment of chronic irreparable patellar tendon injuries. *Knee*. 2020;27(6):1841-1847. doi:10.1016/j.knee.2020.09.002

381. Scott EJ, Willey MC, Mercado A, Davison J, Wilken JM. Assessment of Disability Related to Hip Dysplasia Using Objective Measures of Physical Performance. *Orthop J Sports Med*. 2020;8(2):2325967120903290. Published 2020 Feb 27. doi:10.1177/2325967120903290

382. Harold RE, Butler BA, Delagrammaticas D, Sullivan R, Stover M, Manning DW. Patient-Reported Outcomes Measurement Information System Correlates With Modified Harris Hip Score in Total Hip Arthroplasty. *Orthopedics*. 2021;44(1):e19-e25. doi:10.3928/01477447-20201202-02

383. Ploetze K, Goldfarb C, Roberts S, Wall L. Radiographic and Clinical Outcomes of the Shoulder in Long-Term Follow-Up of Brachial Plexus Birth Injury. *J Hand Surg Am*. 2020;45(12):1115-1122. doi:10.1016/j.jhsa.2020.09.001

384. Kator J, Aneizi A, Nadarajah V, et al. Predictors of early postoperative pain interference following orthopaedic surgery. *J Orthop*. 2020;22:579-583. Published 2020 Nov 13. doi:10.1016/j.jor.2020.11.009

385. Katakam A, Collins AK, Sauder N, et al. Obesity Increases Risk of Failure to Achieve the 1-Year PROMIS PF-10a Minimal Clinically Important Difference Following Total Joint Arthroplasty. *J Arthroplasty*. 2021;36(7S):S184-S191. doi:10.1016/j.arth.2020.11.004

386. Yagnik GP, Santos ED, Rothfeld AA, Uribe JW, Cohn TM. Spanish translation and cross-language validation of the American Shoulder and Elbow Surgeons Standardized Shoulder Assessment Form. *J Shoulder Elbow Surg*. 2021;30(1):151-157. doi:10.1016/j.jse.2020.05.020

387. Tyser AR, Allen CM, Presson AP, et al. Evaluating the performance of PROMIS and QuickDASH instruments in an intercollegiate Division 1 athlete population. *J Shoulder Elbow Surg*. 2021;30(1):158-164. doi:10.1016/j.jse.2020.05.008

388. Rabah NM, Khan HA, Levin JM, Winkelman RD, Mroz TE, Steinmetz MP. The association between patient rating of their spine surgeon and quality of postoperative outcome [published online ahead of print, 2020 Dec 18]. *J Neurosurg Spine*. 2020;1-7. doi:10.3171/2020.7.SPINE20478

389. Jenkins NW, Parrish JM, Nolte MT, Hrynewycz NM, Brundage TS, Singh K. Validating the VR-12 Physical Function Instrument After Anterior Cervical Discectomy and Fusion with SF-12, PROMIS, and NDI. *HSS J*. 2020;16(Suppl 2):443-451. doi:10.1007/s11420-020-09817-w

390. Jenkins NW, Parrish JM, Hrynewycz NM, Brundage TS, Singh K. Longitudinal Evaluation of Patient-Reported Outcomes Measurement Information System for Back and Leg Pain in Minimally Invasive Transforaminal Lumbar Interbody Fusion. *Neurospine*. 2020;17(4):862-870. doi:10.14245/ns.1938398.199

391. Nguyen C, Kortlever JTP, Gonzalez AI, Ring D, Brown LE, Somogyi JR. Attempts to Limit Censoring in Measures of Patient Satisfaction. *J Patient Exp*. 2020;7(6):1094-1100. doi:10.1177/2374373520930468
